# HMGB1 Is Involved in IFN-α Production and TRAIL Expression by HIV-1-Exposed Plasmacytoid Dendritic Cells: Impact of the Crosstalk with NK Cells

**DOI:** 10.1371/journal.ppat.1005407

**Published:** 2016-02-12

**Authors:** Héla Saïdi, Marlène Bras, Pauline Formaglio, Marie-Thérèse Melki, Bruno Charbit, Jean-Philippe Herbeuval, Marie-Lise Gougeon

**Affiliations:** 1 Institut Pasteur, Antiviral Immunity, Biotherapy and Vaccine Unit, Infection and Epidemiology Department, Paris, France; 2 Chemistry and biology, nucleoside and immunology for therapy CBNIT, CNRS, UMR8601, Laboratoire de Chimie et Biochimie Pharmacologiques et Toxicologiques, Université Paris Descartes, PRES Sorbonne Paris Cité, CICB Paris, Paris, France; National Institutes of Health, UNITED STATES

## Abstract

Plasmacytoid dendritic cells (pDCs) are innate sensors of viral infections and important mediators of antiviral innate immunity through their ability to produce large amounts of IFN-α. Moreover, Toll-like receptor 7 (TLR7) and 9 (TLR9) ligands, such as HIV and CpG respectively, turn pDCs into TRAIL-expressing killer pDCs able to lyse HIV-infected CD4^+^ T cells. NK cells can regulate antiviral immunity by modulating pDC functions, and pDC production of IFN-α as well as cell–cell contact is required to promote NK cell functions. Impaired pDC-NK cell crosstalk was reported in the setting of HIV-1 infection, but the impact of HIV-1 on TRAIL expression and innate antiviral immunity during this crosstalk is unknown. Here, we report that low concentrations of CCR5-tropic HIV-1_Ba-L_ promote the release of pro-inflammatory cytokines such as IFN-α, TNF-α, IFN-γ and IL-12, and CCR5-interacting chemokines (MIP-1α and MIP-1β) in NK-pDCs co-cultures. At high HIV-1_BaL_ concentrations, the addition of NK cells did not promote the release of these mediators, suggesting that once efficiently triggered by the virus, pDCs could not integrate new activating signals delivered by NK cells. However, high HIV-1_BaL_ concentrations were required to trigger IFN-α-mediated TRAIL expression at the surface of both pDCs and NK cells during their crosstalk. Interestingly, we identified the alarmin HMGB1, released at pDC-NK cell synapse, as an essential trigger for the secretion of IFN-α and IFN-related soluble mediators during the interplay of HIV-1 exposed pDCs with NK cells. Moreover, HMGB1 was found crucial for mTRAIL translocation to the plasma membrane of both pDCs and NK cells during their crosstalk following pDC exposure to HIV-1. Data from serum analyses of circulating HMGB1, HMGB1-specific antibodies, sTRAIL and IP-10 in a cohort of 67 HIV-1^+^ patients argue for the *in vivo* relevance of these observations. Altogether, these findings identify HMGB1 as a trigger for IFN-α-mediated TRAIL expression at the surface of pDCs and NK cells, and they suggest a novel mechanism of innate control of HIV-1 infection.

## Author Summary

Plasmacytoid dendritic cells (pDC) are the most potent IFN-α-producing cells and serve as an essential link between innate and adaptive immunity. Exposure of pDCs to HIV-1 triggers IFN-α production, which in turn upregulates TNF-related apoptosis-inducing ligand (TRAIL), turning pDCs into killer pDCs, able to kill infected CD4^+^ T cells. At sites of infection, pDCs might activate or get activated by Natural killer (NK) cells, and pDC-NK cell-cell contact is required to promote the cytolytic potential of NK cells. Functional defects in the pDC and NK cell compartments were reported in the setting of HIV-1 infection, but the precise mechanisms by which HIV impairs NK cell and pDC crosstalk remain to be fully elucidated. To address this question, we developed an *ex-vivo* model of NK-pDC interaction, based on a short-term contact between sorted peripheral NK cells and purified pDCs exposed to HIV-1_BaL_. We found that the concentration of HIV-1 is critical to sustain the functional activation of both pDCs and NK cells. Moreover, we identified the alarmin HMGB1 as an essential trigger for the secretion of IFN-α and IFN-related soluble mediators during the interplay of HIV-1-exposed pDCs and NK cells. HMGB1 was also found crucial for HIV-1-induced translocation of TRAIL on both pDC and NK cell membrane. The *in vivo* relevance of the interdependency between HMGB1, IFN- and TRAIL is suggested by the strong positive correlations between circulating levels of these mediators in a cohort of 67 HIV-1 infected patients. Altogether these findings highlight a new function for HMGB1 and they suggest a novel mechanism of innate control of HIV infection.

## Introduction

The innate immune response to infection serves as the first line defense against incoming pathogens and is essential for shaping the quality of the ensuing adaptive immune response [1] [2]. A unique subset of myeloid cells, dendritic cells (DCs), mediate the link between innate and adaptive immunity [3] [4]. DCs include myeloid DCs (mDCs) that are immune sentinels involved in the recognition of pathogens, antigen-presentation and initiation of T-cell immunity in lymphoid organs, and production of proinflammatory cytokines in response to a variety of stimuli [5], and plasmacytoid DCs (pDCs) that secrete high amounts of interferon-α (IFN-α), and initiate the antiviral immune response [6, 7]. Different studies have highlighted an important immunoregulatory role of the interaction of DCs with several other cells of the innate immune system, in particular natural killer (NK) cells [8]. Indeed, during innate responses, NK cells may interact with both pDCs and mDCs and regulate antiviral immunity [9] [10] [11] [12] [13] [14]. Crosstalk between NK cells and mDCs results in activation of both cell types, with DCs triggering NK-cell proliferation, NK-cell mediated killing of immature DCs (iDCs) (editing process), and NK-dependent DC maturation through the release of TNF-α and IFN-γ [15]. NK cells also interact with pDCs and promote the release of IFN-α in an IL-12-dependent way, which in turn triggers the ability of NK cells to kill iDCs [14].

In the setting of HIV infection, several reports identified both numerical and functional defects in the DC and NK cell compartments [16] [17], and the crosstalk between NK cells and DCs is disrupted. The anergic CD56^-^ NK cells that accumulate during progressive HIV-1 infection are impaired in their ability to promote mDC maturation [18]. Little is known about the effect of HIV-1 infection on the crosstalk between NK cells and pDCs. The response of NK cells to direct IFN-α stimulation is defective in viremic HIV-1-infected patients [19] [20] [21], and reduced amount of IFN-α and TNF-α are produced by CpG-stimulated PBMC from HIV-1-infected patients [19]. A defective NK cell response to IFN-α was also reported in other chronic viral infections, such as HCV infection, and it was proposed to be the consequence of the release by pDCs and hepatocytes of substantial amounts of IFN-α which will preferentially stimulate STAT1 rather than STAT4 phosphorylation, resulting in reduced IFN-γ secretion and upregulation of cytolytic activity. These alterations would result with the inability to eliminate the virus and may contribute to the resistant of some patients to IFN-α-based therapies [22].

IFN-α triggers the up-regulation of the TRAIL apoptotic ligand, thus turning pDCs into Interferon-producing Killer pDCs (IKpDCs), capable of killing HIV-infected and uninfected CD4^+^ T cells through TRAIL activation of the Death receptor 5 (DR5) pathway [23] [24]. TRAIL, a TNF superfamily member, is a proapoptotic ligand which is regulated by type-I IFNs on T cells [25] and dendritic cells [26]. The crucial role of TRAIL in antiviral innate response was shown in the context of EMCV murine infection, in which the control of viral replication in the heart is mediated by TRAIL-expressing NK cells. In this model, the expression of TRAIL on NK cells was dependent on IFN-α/ß signaling [27]. HIV-1 infected patients exhibit higher serum levels of TRAIL than non-infected healthy controls, and serum TRAIL levels correlate with serum HIV viral load [28]. The release of TRAIL during HIV-1 transmission occurs very early at the onset of plasma viremia (*i*.*e*., the eclipse phase) [29], and we reported the expression of TRAIL in lymphoid tissues of both HIV-1 infected individuals and SIV-infected macaques [30]. *In vitro* studies demonstrated that monocytes are the major source of soluble TRAIL production under exposure to HIV-1 [28], and *in vivo* study demonstrated that TRAIL participates to CD4^+^ T cell depletion in HIV-1-infected hu-PBL-NOD-SCID mice [31]. However, it is still debating whether TRAIL-mediated apoptosis contributes to CD4^+^ T cell depletion, the hallmark of HIV disease progression, although HIV-1 infection upregulates *in vivo* DR5 expression on CD4^+^ T cells, which are then prone to TRAIL-mediated apoptosis [28] [32]. These observations suggest that the lack of control of viral replication is responsible for HIV-driven activation of pDCs and prolonged IFN-α production, which can lead to detrimental effects of IFN-α including the upregulation of TRAIL on pDCs thus becoming able to kill uninfected DR5-expressing T cells [24].

We recently reported the crucial role of an alarmin, HMGB1, in the regulation of innate antiviral immunity, in particular during the crosstalk between NK cells and DCs [33]. HMGB1 is an abundant nuclear protein, which acts as a potent pro-inflammatory factor when released extracellularly [34]. The discovery by Wang *et al*. [35], in a mouse model of endotoxaemia, that lipopolysaccharide (LPS)-activated macrophages release HMGB1 and that protection against endotoxin lethality could be obtained by administration of anti-HMGB1 antibodies, revealed that HMGB1 is a proinflammatory mediator able to alert the immune system to tissue damage and to trigger an immediate response. This was confirmed by a study by Yanai *et al*. revealing that HMGB proteins function as universal sentinels for nucleic acids, and the absence of HMGBs in Hmgb1(-/-) mice leads to a defect in type-I interferon and inflammatory cytokines induction by DNA or RNA targeted to activate the cytosolic nucleic-acid sensing receptors, and severely impairs the activation of TLR3, TLR7 and TLR9 by their cognate nucleic acids [36]. Thus, HMGB1 plays a crucial role in TLR9-induced activation by CpG-DNA through its association with CpG-DNA and its receptor RAGE, and this DNA–protein complex preassociates with TLR9 in the ER–Golgi intermediate compartment, thus accelerating the delivery of microbial DNA to TLR9 and leading to the recruitment of the TLR adaptor molecule MyD88 [37]. HMGB1 is essential for DC maturation, migration and Th1 polarization [34] [38] [39] [40]. In particular, during NK-iDC crosstalk, DC-activated NK cells secrete HMGB1, which induces DC maturation and protects DCs from lysis [41].

In the setting of HIV-1 infection, elevated plasma levels of HMGB1 were detected during progressive HIV-1 infection, positively associated with viral replication [42]. Recently, we analyzed the impact of HMGB1 on the fate of HIV-1-infected mDCs, and we showed that exposure of mDCs to the virus makes them resistant to NK-cell mediated killing due to HMGB1-induced up-regulation of the intracellular cell death inhibitors, c-FLIP and c-IAP2. Thus, HMGB1 protects HIV-1-infected mDCs from TRAIL-induced apoptosis by NK cells [33]. In addition, we reported that the interaction between NK cells and HIV-1-infected mDCs results in a dramatic increase in viral replication and proviral DNA expression in DCs. This process is mainly triggered by HMGB1, released both by NK cells and mDCs, and blocking HMGB1 strongly inhibited HIV-1 replication in infected DCs [43].

A single study investigated the impact of HMGB1 on the fate of pDCs, and it reported that HMGB1 and its receptor RAGE are required for pDC maturation, representing a loop that modulates this process [38]. Considering that HMGB1 is a danger signal that triggers innate immunity early after pathogen incoming, that it is required for type-I IFN and proinflammatory response, and that it is produced by activated NK cells, we investigated the possible involvement of HMGB1 during NK-pDC crosstalk and the consequences on the fate and antiviral functions of both NK cells and pDCs in the context of HIV-1 infection.

## Results

### HMGB1 is required for HIV-1-induced pDC maturation

**Fig 1:**
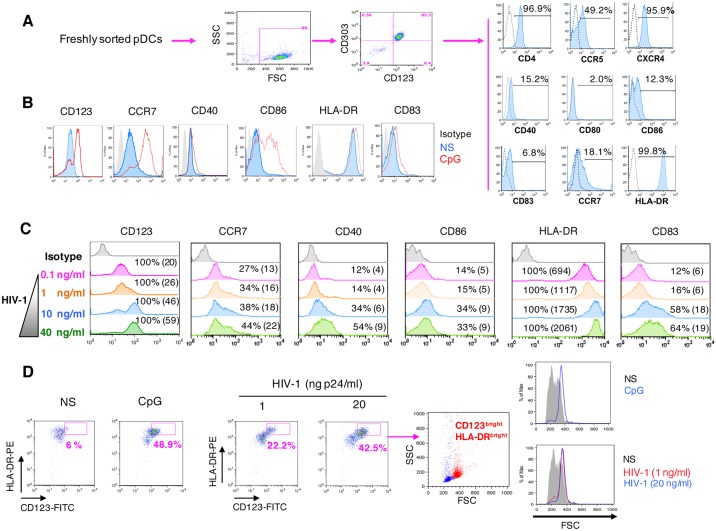
HIV-1 mediates phenotypic maturation of pDCs in a dose-dependent manner. (A): Phenotypic characterization of freshly sorted pDCs. The expression of HIV-1- receptor CD4, co-receptors CCR5 and CXCR4, maturation markers CD40, CD80, CD86, CD83, HLA-DR and chemokine-receptor CCR7 were analyzed by flow cytometry on gated CD123^+^CD303^+^ pDCs. (B): Sorted pDCs were either incubated with medium (NS) or stimulated with CpG (3μg/ml) for 24 hours and the expression of the indicated markers was analyzed. (C): pDCs were exposed to indicated concentrations of HIV-1 and the expression of the indicated markers was analyzed. The percentage of cells expressing the indicated marker and mean fluorescence intensity (in brackets) are indicated. (D): HIV-1-induced phenotypic maturation of pDCs was demonstrated following the identification of mature cells, i.e. CD123^bright^HLA-DR^bright^FSC^high^ cells. These results are representative of at least three different experiments conducted with primary cells from distinct donors.

pDCs were purified from freshly isolated PBMC from healthy donors and their phenotype was characterized. *Ex-vivo* sorted pDCs were all CD123^+^ CD303^+^HLA-DR^+^, and they scarcely expressed CD40, CD80, CD83 and CD86, thus exhibiting the phenotype of immature pDCs. In contrast, CD4 and CXCR4 receptors were expressed in almost all pDCs, while CCR5 was detected in 49% of the population (Fig 1A). TLR-9-dependent stimulation of pDCs with CpG ODN 2006 (3μg/ml) for 24 hours induced their maturation, as shown by the increased expression of CD123, CCR7, CD40, CD86, HLA-DR and CD83 markers (Fig 1B). 18 h-exposure to HIV-1_BaL_ (0.1 to 40 ng p24/ml) induced a dose-dependent maturation of pDCs, evidenced by the increased expression of the fore-mentioned maturation markers (Fig 1C). Notably, the highest HIV-1 concentrations were at least as potent as CpG in inducing CCR7, CD40 and CD83 up-regulation (Fig 1B and 1C). This was confirmed when pDCs were analyzed for the expression of other maturation markers such as CD123 and HLA-DR, mature pDCs exhibiting the CD123^high^HLA-DR^high^ phenotype (Fig 1D). Interestingly, this CD123^high^HLA-DR^high^ mature subset could be easily spotted according to FSC/SSC criteria, corresponding to the red FSC^high^SSC^high^ population on FSC/SSC dot plot, in contrast to the immature pDCs, which appears in blue on FSC/SSC dot plot (Fig 1D). Data in S1 Fig confirm that, in response to diverse stimuli, pDC maturation is associated with the emergence of a specific FSC^high^SSC^high^ population (Panel A). Analysis of the expression of several maturation markers by FSC^high^SSC^high^ pDCs revealed that CD83, CD86 or CD40 were only partially expressed by mature pDCs in response to CpG (40.9%, 58.1%, 73.3% respectively) (panel B). Therefore, we decided to use the HLA-DR/CD123 combination, the only one able to target the whole population of mature pDCs. Mature CD123^bright^HLA-DR^high^FSC^high^SSC^high^ pDC population was induced by HIV-1 at 1ng/ml and 20 ng/ml, and by CpG as well, as shown on dot plots and histograms (Fig 1D).

**Fig 2:**
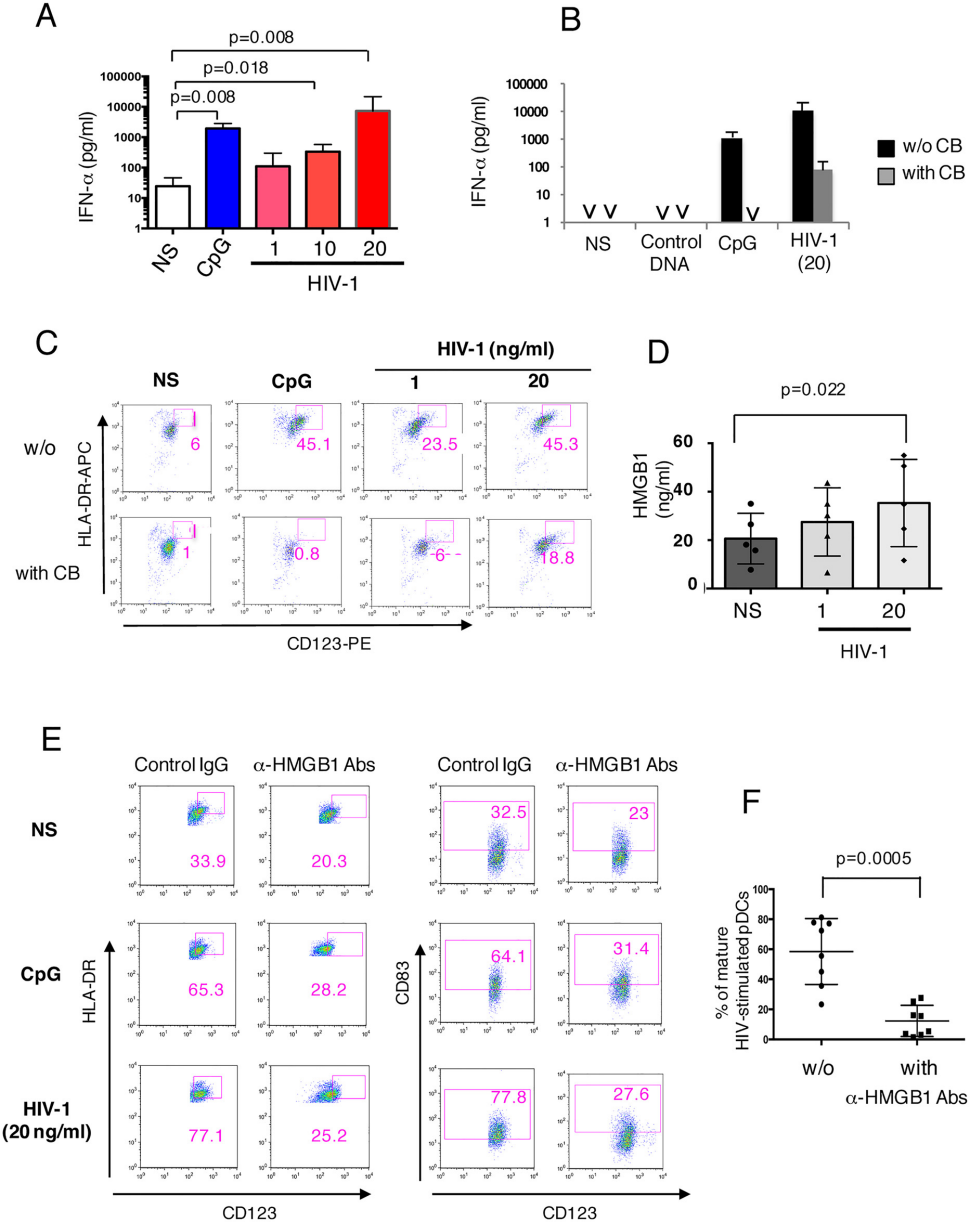
HIV-1-induced phenotypic maturation of pDCs requires HMGB1 and IFN-α. (A): pDCs were exposed to CpG (3 μg/ml), increasing concentrations of HIV-1 (1 to 20 ng/ml), or incubated in medium (NS) during 24 hours. IFN-α concentration in culture supernatants was quantified with a specific ELISA. (B): The IFN-α inhibitor CB was added at 1 μM in cultures of pDCs stimulated with either control DNA (3 μg/ml), CpG (3 μg/ml) or HIV-1 (20 ng/ml p24), and IFN-α concentration in culture supernatants was quantified with a specific ELISA. (C): Inhibition by the IFN-α inhibitor CB (1 μM) of pDC maturation triggered by HIV-1 (1 or 20 ng/ml p24) or CpG (3 μg/ml). Mature pDCs were identified as CD123^bright^HLA-DR^bright^ cells. Results from one representative experiment out of four conducted with primary cells from distinct donors are shown. (D): HMGB1 concentrations were measured in culture supernatants of pDCs exposed 24 hours to the indicated concentrations of HIV-1 with a specific ELISA. (E and F): Impact of neutralizing anti-HMGB1 antibodies (α-HMGB1 Abs) (10 μg/ml) or control IgG on pDC maturation following their culture in medium (NS) or their stimulation with CpG (3μg/ml) or HIV-1 (20 ng/ml) for 24 hours. Mature pDCs were identified as CD123^bright^HLA-DR^bright^ or CD123^+^ CD83^+^ cells. Results from one representative experiment out of four conducted with primary cells from distinct donors are shown. Data shown in Figures A, B, D and F represent the mean values ± SD of at least 4 different experiments performed with distinct donors. Nonparametric Wilcoxon signed-rank test was used to compare the levels of IFN-α or HMGB1 released by pDCs with a p-value ≤ 0.05 being considered as significant.

Viral stimulation triggers pDCs to produce vast amounts of antiviral IFN-α [44] [45]. Accordingly, increasing concentrations of HIV-1 induced a dose-dependent IFN-α production by sorted pDCs (Fig 2A), HIV-1 at 20 ng/ml being as efficient as CpG. We also used clobenpropit (CB), a synthetic histamine analogue [46], to inhibit IFN-α production by pDCs [47]. It was found to be a strong inhibitor of IFN-α release by pDCs in response to both CpG and HIV-1, thus inducing a 3-log decrease in IFN-α concentration in pDCs supernatants (Fig 2B). Concomitantly, CB strongly inhibited pDC maturation induced by CpG or HIV-1, mature pDCs being identified as CD123^high^HLA-DR^high^ cells (Fig 2C). HMGB1 was reported to be required for the maturation of human pDCs upon triggering of TLR9 by CpG, but not following LPS stimulation [38] and, in response to CpG, pDCs relocate and secrete HMGB1 [38]. We show herein that HIV-1 also triggers HMGB1 secretion by pDCs, in a dose-dependent manner (Fig 2D), and neutralizing HMGB1 antibodies strongly inhibit pDC maturation, whether induced by CpG or HIV-1 (20 ng/ml) (Fig 2E), thus reducing significantly (p = 0.0005) the proportions of mature CD123^high^HLA-DR^high^ CD83^+^pDCs (Fig 2F). These data indicate that HMGB1 is required for HIV-1-induced pDC maturation.

### pDCs exposed to HIV-1 produce a broad array of proinflammatory molecules

**Fig 3:**
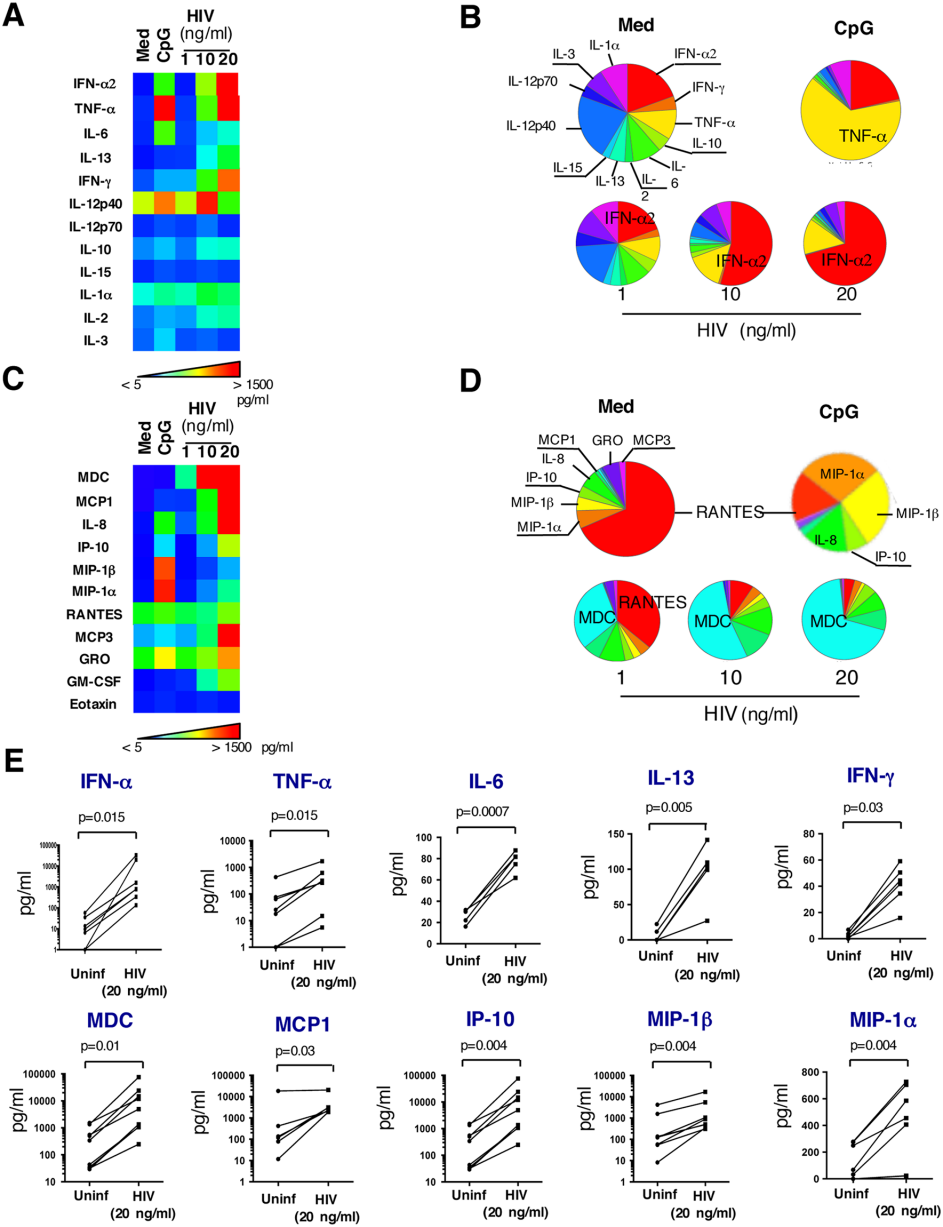
HIV-1 increases the release of proinflammatory cytokines and chemokines by pDCs in a dose-dependent manner. Cytokines and chemokines content were quantified by MAP technology in 24 hour cell-free culture supernatants of pDCs incubated in medium, stimulated with CpG ((3μg/ml) or were exposed to increasing concentrations of HIV-1 (1, 10 and 20 ng/ml p24). Heat-maps (A-C) and Pie Charts (B-D) were used to visualize the broad array of cytokines (A-B) and chemokines (C-D). Analysis of the data was made using SPICE Software. Pictures from one representative experiment out of seven conducted with different primary cell preparations are shown. The mean values ± SD of seven independent experiments testing the impact of HIV-1 (20 ng/ml) stimulation on cytokine and chemokine release by pDCs are shown (E). A p-value ≤ 0.05 was considered significant; non-parametric Wilcoxon test was used.

In addition to type I IFNs, pDCs secrete substantial amounts of other inflammatory molecules. Specifically, it has been shown that pDC stimulation with enveloped viruses, such as herpes simplex or influenza, results in the production of the inflammatory cytokines TNF-α and IL-6, as well as ß chemokines such as MIP-1α and MIP-1ß [48] [49] [50]. However, these studies did not define the extended array of molecules simultaneously produced by pDCs. Such an analytical approach, using multianalyte profiling, was recently used to define the key chemokines and cytokines produced by pDCs in response to TLR-7 and -9 agonists [51]. This approach was not used yet to characterize the pattern of cytokines and chemokines released by pDCs following exposure to HIV. We report herein the extended array of proinflammatory mediators produced by pDCs stimulated with increasing concentrations of HIV-1 (Fig 3). In addition to a dose-dependent increase in IFN-α production (Fig 3A) (in agreement with recently published data [52]), HIV-1 induced the release of other proinflammatory cytokines i.e. TNF-α, IL-6, IL-13, IFN-γ and IL-12 p40 (Fig 3A). HIV-1 at 20 ng/ml was particularly potent in stimulating IFN-α, TNF-α and IFN-γ responses (Fig 3A). To investigate the influence of HIV-1 exposure of pDCs on cytokine microenvironment, the relative proportions of cytokines induced by the virus were compared to those induced by CpG. Fig 3B clearly shows that increasing concentrations triggered an important IFN-α response, which was dominant for HIV-1 at 20 ng/ml, while CpG triggered a dominant TNF-α response. The array of chemokines produced upon pDC activation was also determined (Fig 3C). The great majority of chemokines tested were induced by HIV-1 (i.e. MDC, MCP-1, IL-8, IP-10, MIP-1α, MIP-1β, MCP3, GRO and GM-CSF), in a dose-dependent way. When the relative proportions of chemokines induced by HIV-1 were compared to those induced by CpG, a dominant MDC response was detected upon exposure of pDCs to increasing doses of HIV-1, while the chemokine response to CpG was spread into MIP-1α, MIP-1β, IP-10, IL-8 and RANTES (Fig 3D). Notably, RANTES, a potent HIV suppressive factor [53] was spontaneously produced by pDCs incubated in culture medium, and its relative proportion among chemokines strongly decreased following pDC exposure to increasing doses of HIV-1 (Fig 3D). These experiments were repeated on pDCs from seven healthy donors and the pattern of cytokines and chemokines whose mean levels were significantly increased by HIV-1 at 20 ng/ml is depicted in Fig 3E. These data describe for the first time the broad array of inflammatory mediators produced by HIV-1-stimulated pDCs. Interestingly, the array of chemokines induced by HIV-1-stimulated pDCs overlap the one reported at the mRNA level in herpes simplex virus-stimulated pDCs, and shown to induce migration of activated T cells and NK cells in chemotaxis assays [48].

### Impact of aNK cells on HIV-1-triggered inflammatory response of pDCs

**Fig 4:**
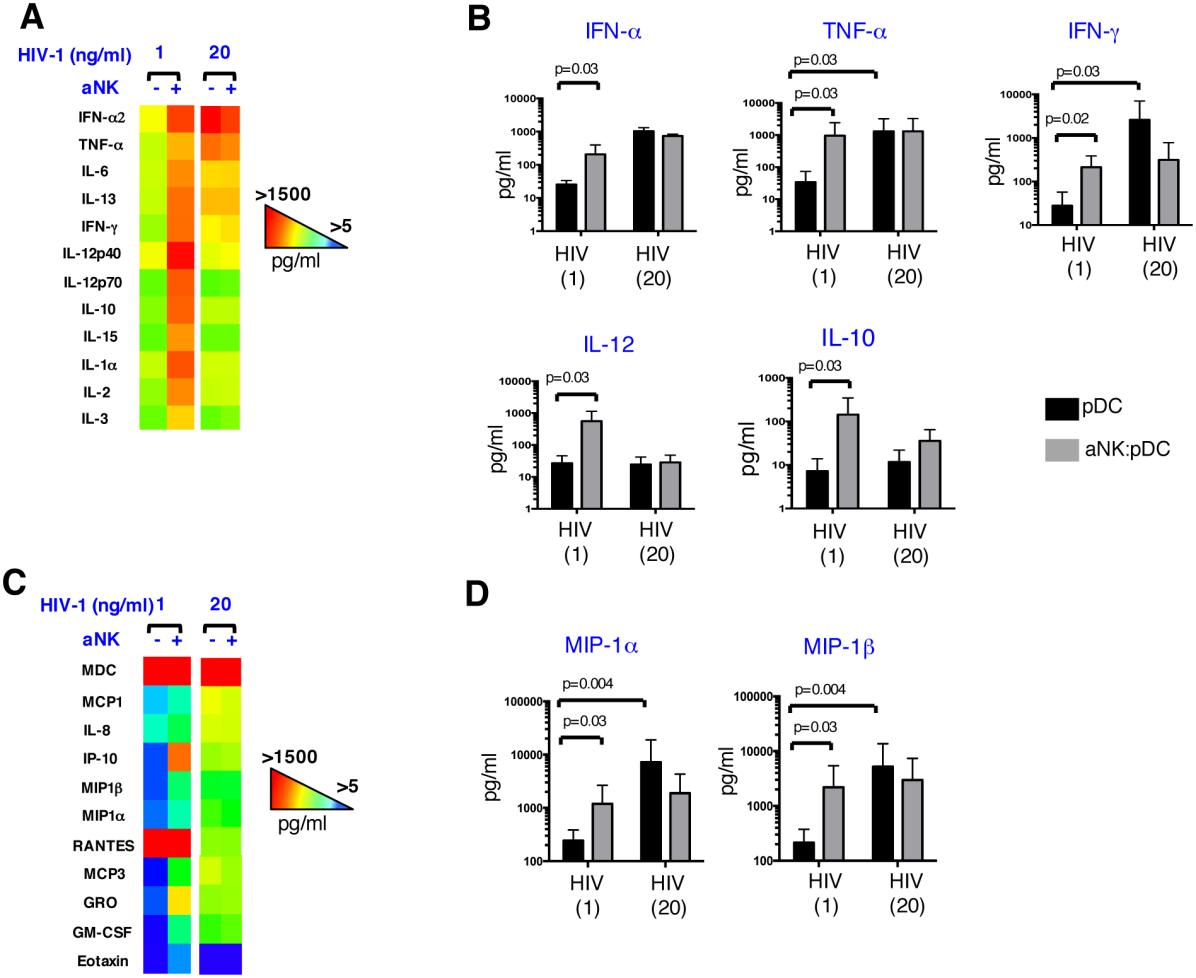
Impact of aNK cells on the proinflammatory array of cytokines and chemokines released by pDCs in the context of HIV-1 infection. Cytokines (A, B) and chemokines (C, D) content were quantified by MAP technology in 24 hour cell-free culture supernatants of pDCs exposed to low (1 ng p24/ml) or high (20 ng p24/ml) concentration of HIV-1, and further cocultured or not with aNK cells. Heat-maps (A, C) from one representative experiment out of four conducted with different primary cell preparations are shown. Mean values ± SD for indicated cytokines and chemokines from six independent experiments are shown (B, D) (non-parametric Wilcoxon test).

The production of soluble mediators during NK-pDC crosstalk was compared to that of aNK cells or pDCs alone (S2 Fig). In our conditions, levels of IL-12, TNF-α or IFN-γ were not detectable in supernatants of pDCs or aNK cells cultivated alone during the short-term culture, while their crosstalk led to the production of significant levels of these inflammatory cytokines. An increased production of IL-8 was also detected in NK-pDC cocultures, as compared to aNK or pDCs alone, while MIP1-α and MIP-1β chemokines were already produced by aNK cells and not increased when cocultured with pDCs. When analyzing the impact of various concentrations of HIV-1 on the level of the inflammatory response generated during NK-pDC crosstalk, we discovered that pDCs exposure to HIV-1 at 1 ng/ml for 3 hours prior to their interaction with aNK cells (ratio 1:5) led to a strong increase in the production of all cytokines tested (n = 11), as compared to the levels of cytokines produced in the absence of aNK cells (Fig 4A). Statistical comparison of mean values obtained for each cytokine from seven experiments performed with cells from independent healthy donors showed that the interaction of pDCs exposed to HIV-1 at 1 ng/ml with aNK cells triggered significant increased levels of IFN-α, TNF-α, IFN-γ, IL-12 and IL-10 (Fig 4B). In contrast, when pDCs were exposed to a higher dose of HIV-1 (20 ng/ml p24), their crosstalk with aNK cells had no impact on the levels of cytokines released (Fig 4A and 4B). Similar observations were made regarding the pattern of chemokines produced in aNK-pDC cocultures exposed to the two different doses of HIV-1 (Fig 4C and 4D). Thus, the majority of chemokines tested i.e. IL-8, IP-10, MIP-1α, MIP-1β, MCP3, GRO and GM-CSF were increased in aNK-pDC cocultures after pDC exposure to HIV-1 at 1 ng/ml, and statistical significant differences were found for MIP-1α and MIP-1β levels, while the chemokine levels were unchanged after exposure of pDCs to HIV-1 at 20 ng/ml prior to coculture with aNK cells. These findings show that the inflammatory microenvironment generated when pDCs interact with aNK cells changes depending on the concentrations of HIV-1 that stimulate pDCs, and they also reveal that once pDCs reach a certain activation threshold in response to HIV-1, they cannot exceed this threshold when interacting with aNK cells.

### HIV-1 is an inducer of HMGB1/RAGE autocrine loop that is involved in pDC maturation and IFN-α secretion during NK-pDC interaction

**Fig 5:**
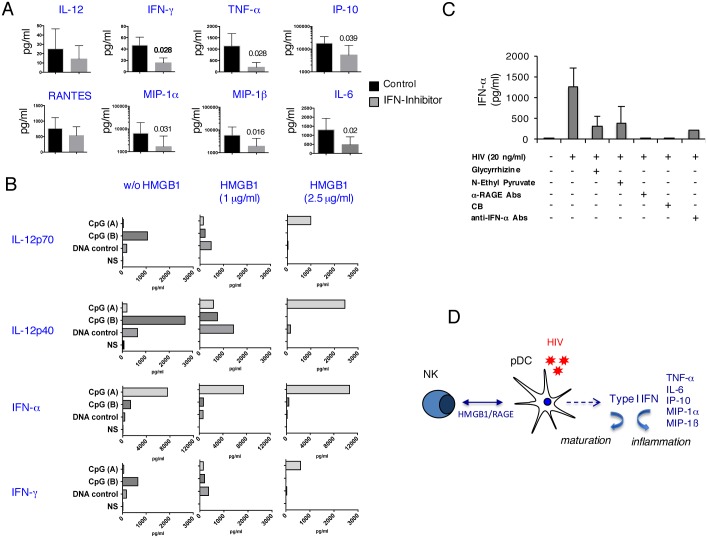
Triggering by HIV-1 of HMGB1/RAGE autocrine loop during NK-pDC interaction. (A): Cytokines and chemokines content were quantified by MAP technology in 24 h cell-free culture supernatants of NK-pDCs co-cultures after exposure of pDCs to HIV-1 (20 ng p24/ml). The IFN-α inhibitor CB (1μM) was added at the initiation of the coculture. The mean values ± SD from three independent experiments are shown. The asterisks indicate statistically significant values (p <0.05) (non-parametric Wilcoxon test). (B): IL-12p70, IL-12p40, IFN-α and IFN-γ were quantified by MAP technology in 24 h cell-free culture supernatants of pDCs stimulated with control DNA, CpG (A) or CpG (B) (3μg/ml) in the absence or presence of two concentrations of recombinant HMGB1 (1 and 2.5 μg/ml). (C): pDCs exposed or not to HIV-1 (20 ng p24/ml) were cultured for 24 h with aNK cells, and a number of inhibitors were added at the initiation of the coculture: glycyrrhizin at 10μg/ml, N-ethyl pyruvate at 10μM, anti-RAGE antibodies at 10 μg/ml, the IFN-α inhibitor CB at 1μM, and anti-IFN-α antibodies IFN-α at 10 μg/ml. IFN-α was quantified by specific ELISA in 24 h cell-free culture supernatants. Histograms show the mean values ± SD (triplicates) from one representative experiment, out of three independent experiments. (D): A schematic representation illustrating the requirement for HMGB1/RAGE autocrine loop for triggering NK-mediated activation of pDCs and IFN-α production in the context of HIV-1 infection.

HIV-1 triggers the production of IFN-α by pDCs [54], which also produce proinflammatory cytokines, such as TNF-α, IFN-γ or IL-6, and chemokines, including IP-10, MIP-1α, and MIP-1β (Fig 3) [48] [55]. In further dissecting the mechanisms by which pDCs initiate inflammatory responses and defining IFN-α-driven loops triggered by viral stimulation, we used CB, a potent inhibitor of IFN-α release, as demonstrated in Fig 2B. As shown in Fig 5A, pDC-derived IFN- α mediates the release of IL-12, IFN-γ, TNF-α, IL-6, IP-10, MIP-1α, and MIP-1β after HIV-1 stimulation, as blocking IFN-α release by CB significantly inhibited the expression of these aforementioned mediators. We showed above that TLR stimuli (HIV-1 and CpG) induced in pDCs the production of a broad array of inflammatory mediators (Fig 3), and also the release of HMGB1 (Fig 2). To investigate how HMGB1 contributes to the initiation by pDCs of an inflammatory milieu, pDCs were stimulated with ODN 2216 (CpG-A) or ODN 2006 (CpG-B), potent and weak inducer of IFN-α respectively [56], in the presence of two concentrations (1μg/ml and 2.5μg/ml) of exogenous rHMGB1. Data in Fig 5B show that, as expected, CpG-B induced the release of low levels of IFN-α while it triggered the production of IL-12 and IFN-γ. Addition of exogenous rHMGB1 at 2.5 μg/ml completely abrogated the release of these two cytokines (Fig 5B). In contrast, CpG-A induced in pDCs a potent IFN-α response, while the other cytokines (IL-12 and IFN-γ) were not detected. The addition of exogenous rHMGB1 to CpG-A induced a strong inflammatory response, characterized by increased levels of IFN-α and the production of IL-12p70, IL-12p40 and IFN-γ (Fig 5B). Such a stimulating effect of HMGB1 was not observed on CpG-B-stimulated pDCs (Fig 5B). These findings are consistent with previous observations showing that CpG-A, but not CpG-B, augments the binding of HMGB1 to its receptor RAGE, resulting in a considerable increased inflammatory cytokine production by mean of TLR9 and RAGE [37]. Thus, exogenous HMGB1 strongly contributes to the initiation by pDCs of the inflammatory milieu.

As we discovered the pivotal role of HMGB1 in promoting HIV dissemination and latency in mDCs during mDC-NK crosstalk [43] [33] [57], we decided to decipher the role of HMGB1 in the context of NK-pDC interaction. aNK cells were cocultured with pDCs (aNK:pDC ratio 1:5) previously exposed to HIV-1 at 20 ng/ml p24, and the influence of HMGB1 antagonists on IFN-α response of pDCs was determined. Glycyrrhizin, which binds to HMGB1 and inhibits its cytokine activity [58], strongly inhibited IFN-α release by HIV-1 exposed pDCs during their interaction with aNK cells (Fig 5C). N-ethyl pyruvate that inhibits HMGB1 release by preventing its nuclear-to-cytoplasmic translocation [59] was also a potent inhibitor of IFN-α response in the same cultures. Similar effect was obtained with antibodies specific for RAGE, one of the HMGB1 receptors, which totally suppressed IFN-α response to HIV-1 (Fig 5C). As expected, IFN-α Inhibitors, such as anti-IFN-α antibodies or CB, abrogated the IFN-α response of HIV-exposed pDCs during their interaction with aNK cells (Fig 5C). These findings report for the first time that HIV-1 is an inducer of HMGB1/RAGE autocrine loop that is involved in pDC maturation and IFN-α secretion during NK-pDC interaction, thus promoting an inflammatory milieu (Fig 5D). pDC response to TLR9 stimulus was reported to involve such an autocrine loop [38].

### HIV-1 triggers mTRAIL expression on both pDCs and NK cells during NK-pDC crosstalk

**Fig 6:**
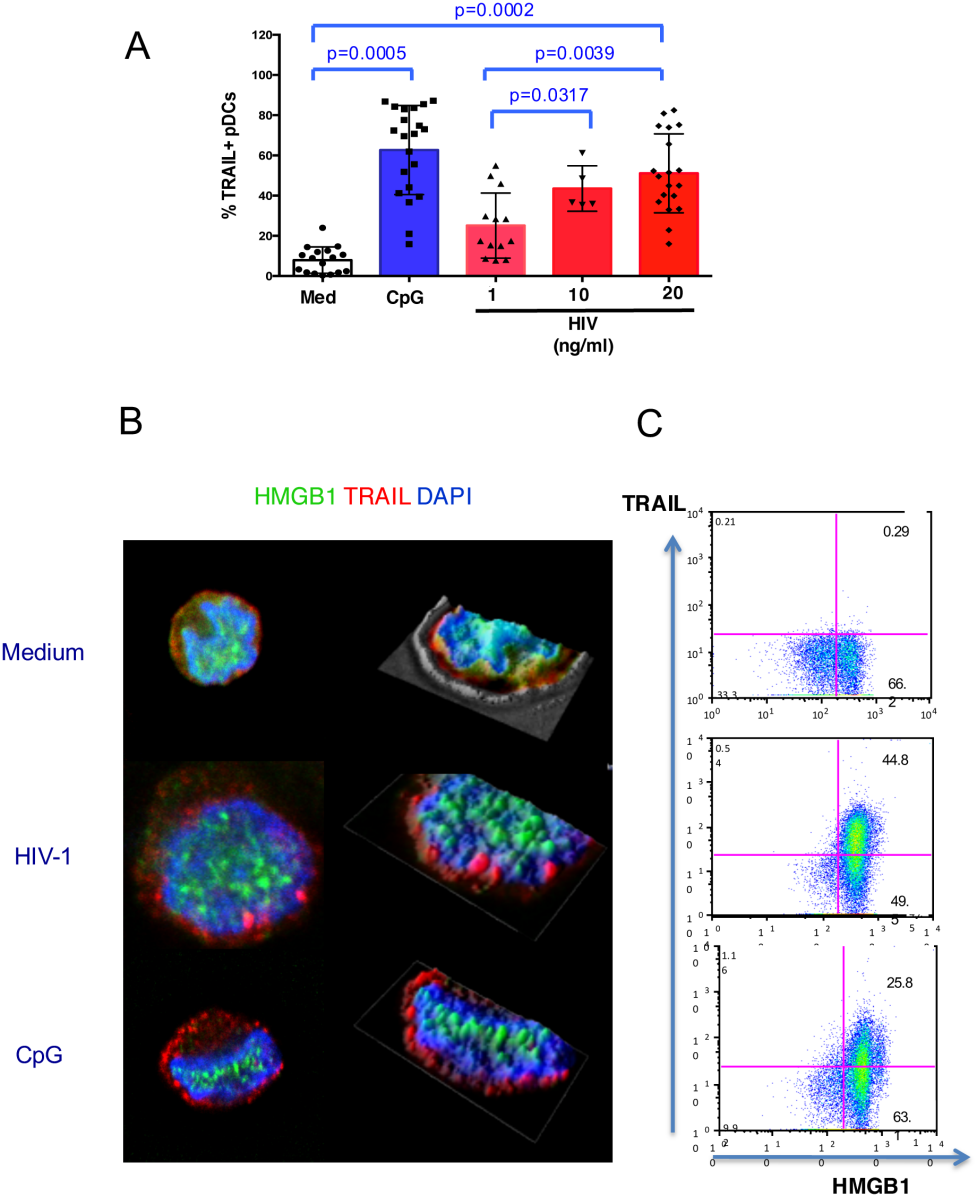
HIV-1 triggers the translocation of TRAIL from the cytoplasm to the membrane of pDCs. (A): pDCs were exposed to CpG (3 μg/ml) or increasing concentrations of HIV-1 (1 to 20 ng p24/ml) for 24 h. The expression of mTRAIL at the surface of pDCs was monitored by flow cytometry. The mean values ± SD of five independent experiments are shown (p-value ≤ 0.05 was considered significant; non-parametric Mann-Whitney test). (B): To characterize TRAIL and HMGB1 localization on pDCs exposed to HIV-1 (1ng/ml p24) or CpG (3 μg/ml), images were analysed using 3D interactive surface plot analysis. Deconvolution overlays of representative 2D red cross axis XY focal plan with XZ/YZ view of focal plane and projection overlay of cell stainings (analyzed by Metamorph software) are shown. (C): Flow cytometry analysis of mTRAIL expression and HMGB1 intracellular staining of pDCs cultured in medium, exposed to HIV-1 (1 ng p24/ml) or stimulated with CpG (3 μg/ml). Dot plots from one representative experiment out of three independent experiments are shown.

We previously showed that HIV-1 virions turn pDC into TRAIL-expressing IFN-producing killer pDC (IKpDC) [23]. We also reported that unstimulated pDCs were "dormant" IKpDCs with high levels of intracellular TRAIL that could be rapidly mobilized to cell surface in response to cell-free HTLV-1 [60] or HIV-1 [61]. We show in Fig 6A that 3-hour exposure of pDCs to HIV-1 induces a dose-dependent significant increase of mTRAIL^+^ pDCs (p = 0.0002 comparing pDCs exposed to HIV-1 20 ng/ml vs uninfected pDCs). Notably, TLR-9 ligation by CpG was also a potent inducer of mTRAIL on pDCs (Fig 6A). We performed 3D microscopy experiments and 3D reconstruction analysis to study TRAIL and HMGB1 expression and localization in pDCs stimulated with either HIV-1 or CpG. High levels of intracytoplasmic TRAIL were detected in unstimulated pDCs, associated with nuclear localization of HMGB1 (Fig 6B, upper panel). Following exposure to HIV-1, mTRAIL was translocated at the membrane of pDCs while nuclear HMGB1 staining was patched (Fig 6B, middle panel). Similar images were observed upon pDC activation with CpG **(** Fig 6B, lower panel). Flow cytometry analysis after dual staining (membrane staining with anti-TRAIL antibodies followed by intracellular staining with anti-HMGB1 antibodies) of pDCs submitted to the same stimuli confirm the lack of expression of mTRAIL in unstimulated pDCs, while their exposure to HIV-1 or CpG induced mTRAIL expression in 44.8% and 25.8% of pDCs respectively, all of them expressing intracellular HMGB1, as expected (Fig 6C).

**Fig 7:**
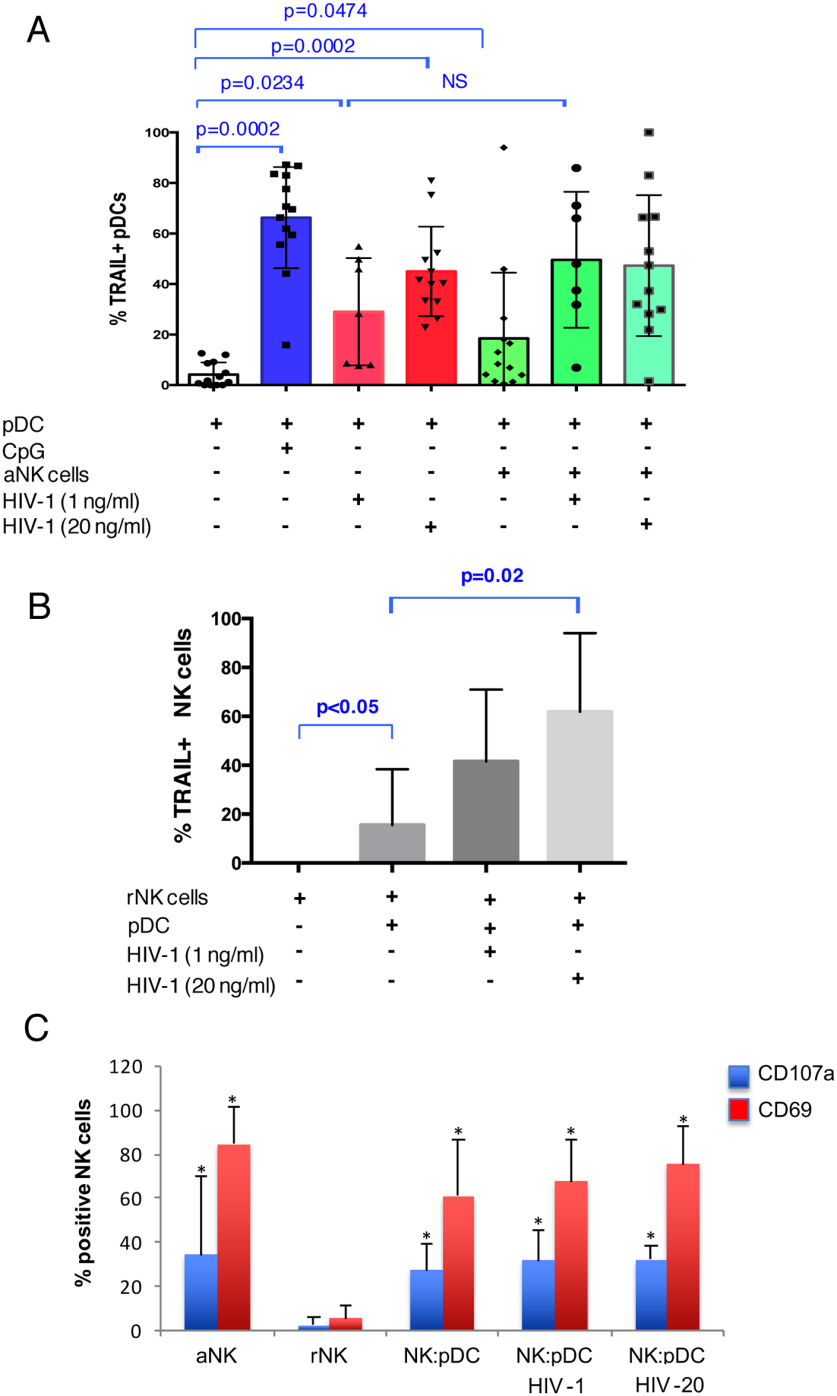
HIV-1 triggers mTRAIL expression on both pDCs and NK cells during NK-pDC crosstalk. (A): pDCs were exposed or not to HIV-1 (1 ng/ml or 20 ng/ml p24) and further cultured for 24 h in the presence or not of aNK cells. Stimulation with CpG (3 μg/ml) was used as positive control. mTRAIL expression at the surface of pDCs was monitored by flow cytometry. (B): Resting NK cells (rNK cells) were cultured alone or with pDCs after their exposure or not to HIV-1 (1 ng/ml or 20 ng/ml). mTRAIL expression at the surface of NK cells was assessed by flow cytometry. The mean values ± SD of seven independent experiments are shown (non-parametric Mann-Whitney test). (C) Resting NK cells (rNK cells) were cultured alone or with pDCs after their exposure or not to HIV-1 (1 ng/ml or 20 ng/ml). The expression of the cytotoxic marker CD107 or the activation marker CD69 was monitored by flow cytometry. The mean values ± SD of seven independent experiments are shown (non-parametric Mann-Whitney test).

The impact of the interaction of HIV-1 exposed pDCs with aNK cells on mTRAIL expression on pDCs was analyzed on cells from twelve healthy donors (Fig 7A). A mean increase in the percentage of mTRAIL^+^ pDCs was detected for the lower concentration of HIV-1 (1 ng/ml p24), although not statistically significant (Fig 7A). For the highest concentration of HIV-1 (20 ng/ml), the interaction of pDCs with aNK cells did not increase the mean percentage of TRAIL^+^ pDCs. Notably, in the absence of HIV-1, aNK:pDC interaction triggered mTRAIL expression on pDCs (p = 0.047 vs pDC). We then addressed the question of pDC-triggered mTRAIL expression on NK cells. Resting NK cells (rNK) were co-cultured for 24 h in the presence of pDCs previously exposed to HIV-1 at 1 ng/ml or 20 ng/ml. As expected, sorted rNK cells did not express mTRAIL. Their interaction with pDCs, in the absence of virus, triggered mTRAIL expression on NK cell surface (p<0.05 vs rNK cells) (Fig 7B). Their interaction with pDCs exposed to HIV-1 triggered a strong and significant increase of mTRAIL expression on rNK cells, reaching for the highest HIV concentration a mean of 60% mTRAIL^+^NK cells (p = 0.02 vs rNK-pDC w/o HIV). Moreover, pDC-triggered acquisition of mTRAIL by rNK cells was accompanied by the expression of the activation marker CD69 and the marker of degranulation CD107a, detected both in the absence and in the presence of HIV-1, and reaching levels comparable to those expressed by aNK cells (Fig 7C). Altogether these observations reveal that, in response to HIV-1, NK-pDC interaction triggers the generation of cytotoxic cells in both interacting subsets, revealed by the expression of the apoptotic ligand TRAIL and also the lytic granule membrane protein CD107a, whose expression is a functional marker of NK cytolytic activity [62].

### HMGB1 triggers mTRAIL expression on both pDCs and NK cells during NK-pDC interaction

**Fig 8:**
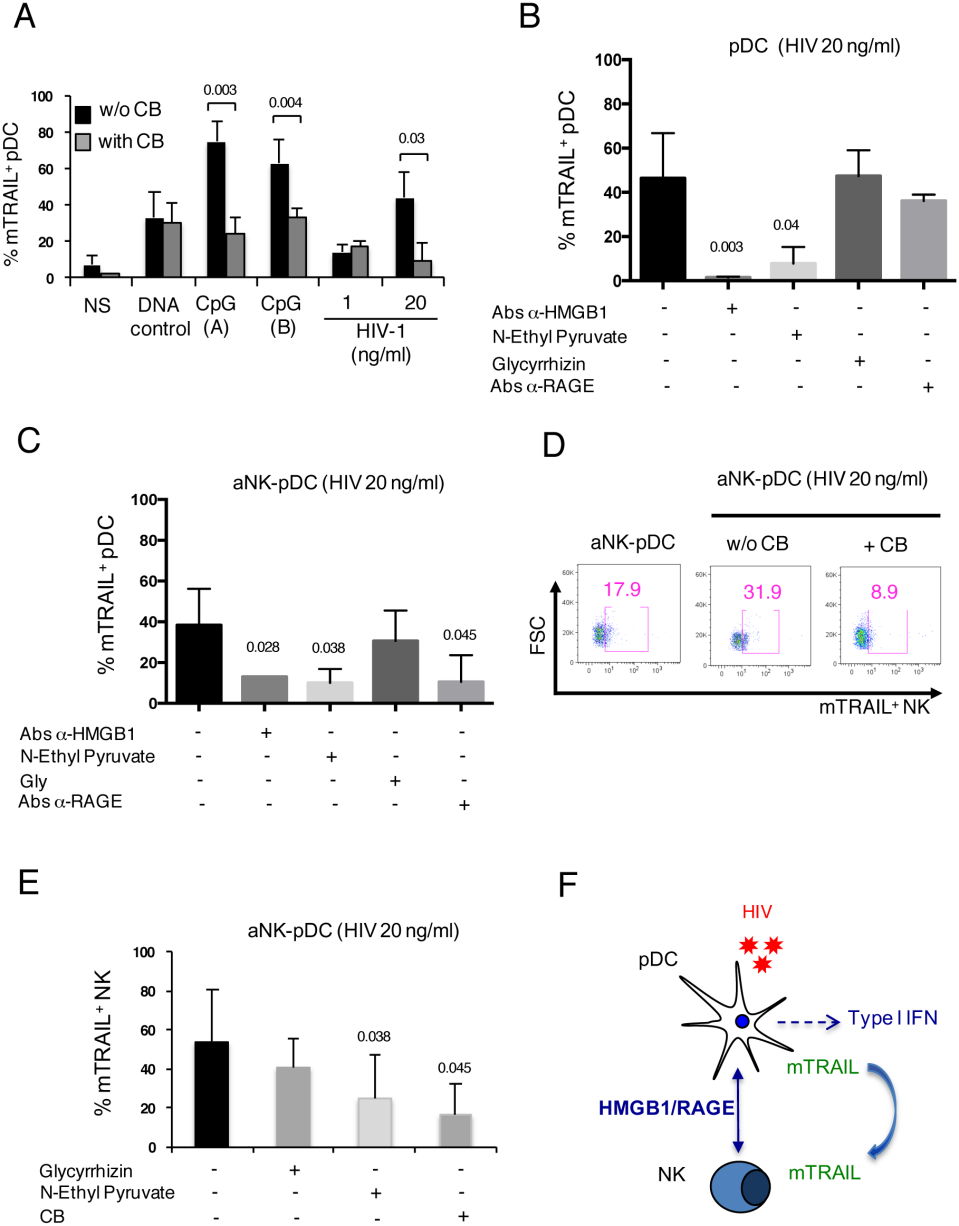
HMGB1 triggers mTRAIL expression on both HIV-1-exposed pDCs and NK cells during their crosstalk. (A): pDCs were stimulated for 24 h with control DNA, CpG (A) or CpG (B) or exposed to HIV-1 at 1 ng/ml or 20 ng/ml. The IFN-α inhibitor CB (1μM) was added at the initiation of the coculture. The expression of mTRAIL at the surface of pDCs was monitored by flow cytometry. Mean values ± SD of three independent experiments (non-parametric Mann-Whitney test). (B): pDCs were exposed to HIV-1 at 20 ng/ml in the presence or not of various inhibitors: anti-HMGB1 antibodies (10 μg/ml), N-ethyl pyruvate (1 μM), glycyrrhizin (10 μg/ml), or anti-RAGE antibodies (10 μg/ml). mTRAIL expression at the surface of pDCs was monitored by flow cytometry. Mean values ± SD of three independent experiments (non-parametric Mann-Whitney test). (C): pDCs exposed to HIV-1 at 20 ng/ml were cocultured with aNK cells in the presence of various inhibitors: anti-HMGB1 antibodies (10 μg/ml), N-ethyl pyruvate (1 μM), glycyrrhizin (10 μg/ml), or anti-RAGE antibodies (10 μg/ml). mTRAIL expression at the surface of pDCs was monitored by flow cytometry. Mean values ± SD of three independent experiments (non-parametric Mann-Whitney test). (D): pDCs exposed or not to HIV-1 at 20 ng/ml were cocultured with aNK cells in the presence or not of the IFN-α inhibitor CB (1μM). mTRAIL expression on gated NK cells was monitored by flow cytometry. Representative dot plots are shown. (E): pDCs exposed to HIV-1 at 20 ng/ml were cocultured with aNK cells in the presence of glycyrrhizin (10 μg/ml), N-ethyl pyruvate (1 μM), and CB (1 μM). Mean values ± SD of three independent experiments (non-parametric Mann-Whitney test). (F) A schematic representation illustrating the requirement for HMGB1/RAGE autocrine loop for the triggering of IFN-α-dependent TRAIL expression at the surface of both pDCs and NK cells in response to HIV-1 infection.

We previously reported that HIV-1-mediated expression of mTRAIL on CD4^+^ T cells [63] and pDCs [23] is regulated by IFN-α. The close link between IFN-α release by pDCs and TRAIL translocation to cell membrane was confirmed using CB, which we showed in Fig 2B to be a strong inhibitor of IFN-α release by pDCs in response to both CpG and HIV-1. As expected, CB significantly inhibited mTRAIL expression on pDCs stimulated either by CpG (A) or CpG (B), or activated by HIV-1 at 20 ng/ml (Fig 8A). Since we found that HMGB1 was required for IFN-α production during NK-pDC crosstalk (Fig 5C), it was of interest to test the possible involvement of HMGB1 on mTRAIL expression on pDCs and NK cells. Data in Fig 8B show that inhibiting HMGB1 with either specific antibodies or N-ethyl pyruvate significantly inhibited the expression of mTRAIL on pDCs exposed to HIV-1 at 20 ng/ml. A weak effect was observed in the presence of anti-RAGE antibodies. In the context of aNK-pDC crosstalk following pDCs exposure to HIV-1 at 20 ng/ml, mTRAIL expression on pDCs was also inhibited by several HMGB1 antagonists, including antibodies specific for HMGB1 or RAGE and N-ethyl pyruvate (Fig 8C). The triggering of mTRAIL expression on NK cells as a consequence of their interaction with HIV-stimulated pDCs required IFN-α release by pDCs, as shown in Fig 8D on dot plots from one representative experiment using CB as an inhibitor. Moreover, inhibiting HMGB1 with N-ethyl pyruvate during NK-pDC interaction significantly decreased the expression of mTRAIL on NK cells (Fig 8E). Altogether, these findings indicate that HMGB1 is essential for the induction of mTRAIL on both pDCs and NK cells in response to HIV-1 (Fig 8F).

### Upregulation of sTRAIL in the blood of HIV-1 infected patients is correlated with HMGB1-specific antibodies, IP10 and HIV-1 proviral DNA

**Fig 9:**
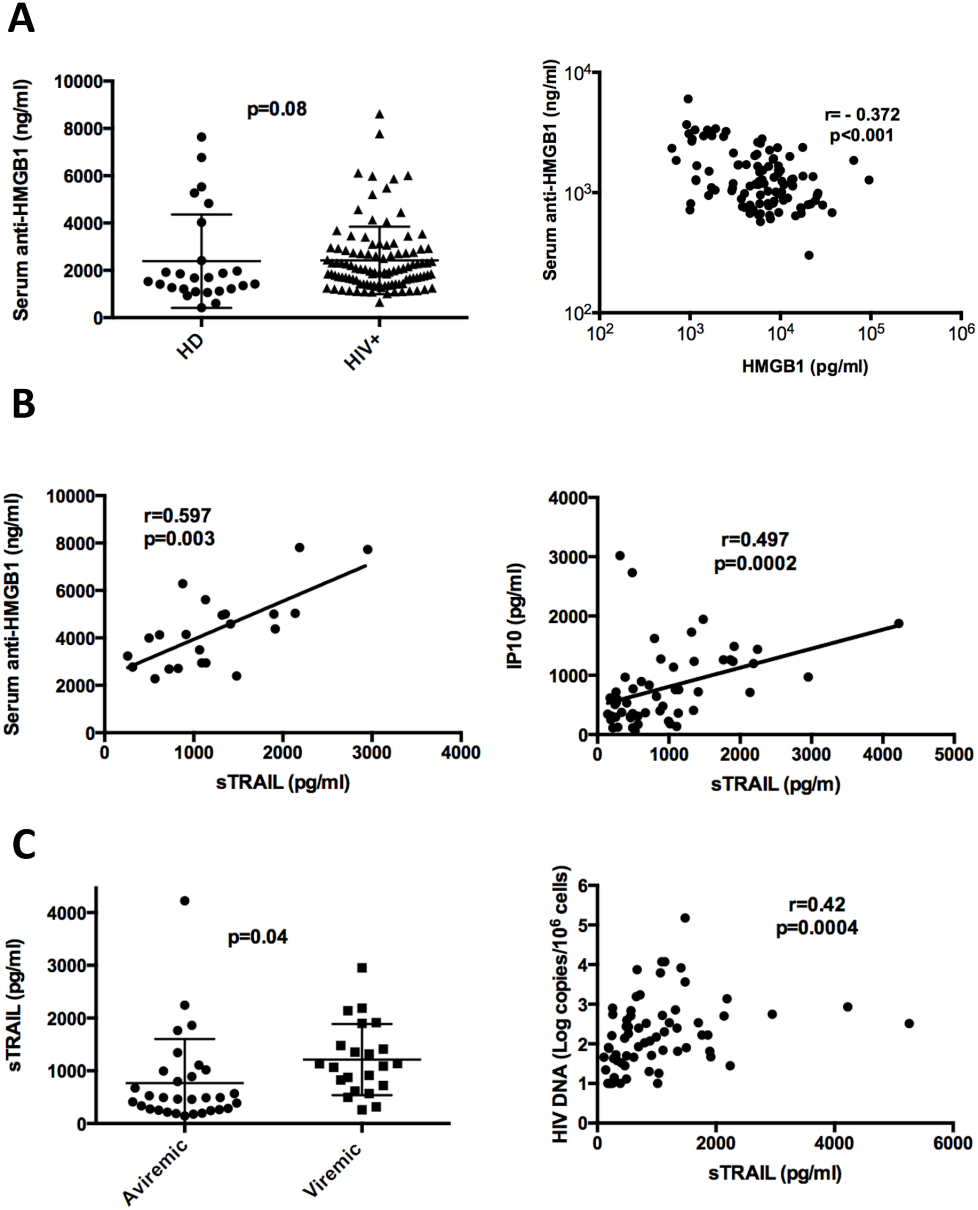
Circulating levels of HMGB1-specific antibodies, sTRAIL, and IP-10 in HIV-1 infected patients. (A): Serum from healthy donors (n = 12) and HIV-1-infected patients (n = 67) were tested for their content in total (residual + HMGB1-bound) HMGB1-specific IgG antibodies with an in-house ELISA assay, as described in M&M. Median and IQR are shown on the left hand side diagram. Spearman correlation between residual HMGB1 and total anti-HMGB1 antibodies is shown on the right hand side. (B): Spearman correlation between sTRAIL and total anti-HMGB1 antibodies in viremic patients (n = 22) on the left hand side, and between sTRAIL and IP-10 (n = 67) on the right hand side. (C): Comparative levels of circulating sTRAIL in aviremic (n = 19) vs viremic (n = 22) HIV-infected patients on the left hand side; spearman correlation between sTRAIL and HIV proviral DNA in the whole group HIV-infected patients (n = 67).

HMGB1 was originally discovered as a 25 kDa DNA-binding protein that participates in many nuclear functions and, triggered by infection and proinflammatory stimuli, HMGB1 can be released extracellularly and act as a proinflammatory mediator. HMGB1 attached to DNA or by itself is highly immunogenic and stimulates the production of autoantibodies [64] [65]. Serum anti-HMGB1 antibodies have been first reported in SLE patients [66], but were also detected in healthy individuals [66] [67]. The HMGB1-binding antibodies might play an important physiological role by modulating the proinflammatory activity of HMGB1, thereby limiting overwhelming inflammatory responses caused by massive HMGB1 release in conditions such as infections or extensive necrosis [68]. It is noteworthy that anti-HMGB1 antibodies, as well as other HMGB1-binding serum proteins, impede the reliable quantification of serum HMGB1 by ELISA, as reported by several groups [69] [66]. Since reliable quantification of circulating HMGB1 remains a problem, we decided to measure instead HMGB1-specific antibodies. We used an in-house ELISA that enables the detection of all HMGB1-specific IgG antibodies i.e. circulating antibodies plus HMGB1-complexed antibodies that have been dissociated from HMGB1 by an acidic treatment (see M&M). Thus the whole range of HMGB1 antibodies that we measure is the direct consequence of the total amount of HMGB1 produced. Data in Fig 9A show that anti-HMGB1 antibodies are detected in serum from healthy donors, and they indicate for the first time that their levels is increased in the setting of HIV-1 infection. An inverse correlation between residual HMGB1 and total anti-HMGB1 IgG antibodies was found, as expected from a putative modulating role of HMGB1-specific antibodies. The level of circulating sTRAIL was assessed in the same cohort of patients and it was strongly correlated with the level of anti-HMGB1 antibodies in viremic patients (r = 0.597, p = 0.003), and with the level of IP10 in the whole group (r = 0.497, p = 0.0002) (Fig 9B). These observations suggest for the first time a link between HMGB1-specific antibodies, sTRAIL and IP-10, whose production by pDCs is strongly triggered by IFNα [70]. The impact of viral load on the levels of circulating sTRAIL in HIV-infected patients is shown in Fig 9C. Increased levels of sTRAIL were detected in viremic patients as compared to aviremic ones, and sTRAIL levels were found strongly correlated with circulating proviral HIV DNA (r = 0.42, p = 0.0004), suggesting that circulating TRAIL might contribute to HIV persistence.

## Discussion

Our study provides evidence that, upon interaction with pDCs, aNK cells promote the release of IFN-α, Th1 cytokines (IL-12 and IFN-γ), CCR5-interacting chemokines (MIP-1α and MIP-1β) and IL-8, in the absence of HIV-1 or upon exposure to low concentration of HIV-1. In contrast, after exposure of pDCs to higher concentration of HIV-1, aNK cells do not promote pDC-dependent production of these mediators. We identified HMGB1, released both by pDCs and NK cells, as being an essential trigger for the secretion of IFN-α and IFN-related soluble mediators (IFN-γ, TNF-α, IP-10, MIP-1α, MIP-1β and IL-6) during the interplay of HIV-1-exposed pDCs with NK cells. In addition, HMGB1 was found crucial for the expression of mTRAIL on both pDCs and NK cell surface. TRAIL translocation on pDC membrane was induced by HIV-1 in a dose-dependent manner, and the presence of NK cells did not modulate this response. However, the crosstalk between HIV-1-exposed pDCs and activated NK cells promoted the expression of TRAIL on NK-cell membrane, which required HMGB1, suggesting that HMGB1 is key element between the two innate immune effectors for the triggering of their cytotoxic functions in response to viral infection.

The host response to viral infection starts when TLRs recognize Pathogen-Associated Molecular Patterns (PAMPs) such as DNA or single strain RNA (ssRNA), then leading to the secretion of proinflammatory mediators. pDCs use TLR9 to detect pathogen-associated DNA (CpG) and to trigger the production of type-I interferon as well as other inflammatory cytokines. Regarding the recognition by pDCs of a ssRNA virus, such as HIV-1, it was shown by Beignon *et al*. that endocytosis of HIV-1 RNA activates TLR7 and results in the production of IFN-α [71]. Accordingly, silencing TLR7 or inhibiting TLR7 pathway greatly reduces IFNα production in HIV-1-infected pDC cell line [72]. We report here that high concentrations of HIV-1, but not newly produced virions, are required to induce the secretion of high levels of IFN-α by pDCs, consistent with the observation that the number of HIV-RNA copies are critical to activate pDCs through TLR signalling [71]. The cytokine and chemokine response induced by HIV-1 probably involves TLR7 pathway, although we cannot exclude the involvement of TLR9 signalling. Indeed, the HIV-1 viral cycle includes the formation of a DNA/RNA heterodimer and a double stranded proviral DNA, which represent potential ligands for TLR9 via CpG-rich DNA regions [73] [74]. It would be interesting to evaluate whether fragments of viral DNA included in the virions engulfed by pDCs and processed by the endosomal pathway could trigger TLR9-mediated responses.

In the absence of virus, we show that aNK cells trigger the release of cytokines and chemokines by pDCs without inducing their phenotypic maturation. These data suggest that NK-mediated induction of pDC activation in the absence of danger signals may be important in the amplification of ongoing inflammatory responses [75] [76]. Strikingly, NK cells were able to efficiently activate pDCs only in the presence of low but not high concentrations of HIV-1. Indeed, the phenotypic and functional properties of pDCs exposed to high concentration of HIV-1 were unchanged following their interaction with NK cells. These observations suggest that, once stimulated by high virus concentration, pDCs could not integrate new activating signals delivered by NK cells. This activation threshold may be triggered by IFN-α produced at high levels under these coculture conditions. This is suggested by studies showing that pDCs exposure to high levels of IFN-α may affect their ability to respond to subsequent de novo stimulation [77], through a negative feedback to limit the extent and duration of IFN-α response. This regulation may involve SOCS proteins that are induced by type I IFNs, and compete with STATs for binding to IFN-α receptor and suppress JAK activity [78] [79]. We cannot completely exclude the involvement of the regulatory cytokines TGFß produced by pDCs exposed to high levels of HIV-1, which could prevent further release of IFN-α and then activation of pDCs [80] [81]. However, this hypothesis is not supported by the very low levels of IL-10 produced during the coculture with aNK cells of pDCs exposed to high virus concentration (Fig 4A and 4B). Regarding the involvement of TGFβ as a suppressor cytokine induced by high virus levels, our data do not support this hypothesis. Indeed, exposure of pDCs to TGFβ was shown to prevent type I interferon secretion [82], whereas high levels of IFN-α produced at high virus concentration. Moreover, TGFβ is known to trigger the production by pDCs of proinflammatory cytokines, such as TNF-α or IL-6, which were not increased during the coculture vs pDCs at high virus concentration (Fig 4A and 4B).

pDCs produce IFN-α after HIV-1 exposure, which in turn regulates TRAIL expression by CD4^+^ T cells, but also by pDCs, thus becoming IKpDCs [23]. We confirmed in the present study that the increase of TRAIL expression on pDC cell surface was due to the translocation of the molecule from the cytoplasm towards cell membrane, as previously shown [61]. We found that TRAIL translocation on pDC membrane was abrogated by the neutralization of IFN-α, suggesting that autocrine IFN-α controls functional activation of pDCs. In contrast to pDCs, exposure of NK cells to high concentrations of HIV-1 did not trigger cell surface expression of TRAIL (S3 Fig). TRAIL translocation on NK-cell membrane required the release of IFN-α by HIV-exposed pDCs, which is consistent with previous observations showing that exogenous NK stimulation derived from pDCs can trigger NK cytotoxicity against HIV-1-infected autologous CD4^+^ primary T cells [83]. It was reported that, following HIV-1 exposure of the vaginal mucosa, pDCs produce β-chemokines that in turn activate the recruitment of CCR5^+^ T cells [84]. This observation suggests that pDCs are activated by the microenvironment or other immune mucosal cells, such as NK cells and myeloid DC. A recent study reported that depletion of NK cells during acute SIV infection consecutive to *in vivo* administration of a JAK3 inhibitor had no impact on plasma viral load in the acute phase but induced an increased viral load in the chronic phase. This treatment also inhibited the redistribution of pDCs from the blood to the GIT, suggesting a protective role of NK-pDC interactions during chronic infection [85].

HMGB1 is abundantly expressed in the nuclei of mammalian cells (>10^6^ molecules/nucleus), facilitating interaction of chromatin with various nuclear proteins [86]. HMGB1 reaches the extracellular environment when innate immune cells secrete it following acetylation [87], either by passive release from necrotic cells [88] or by active release by apoptotic cells [89]. In our experiments, we observed that HIV-1 had a survival effect (increased percentage of Bcl2^high^ cells) rather than a killing effect on pDCs (S4 Fig), excluding that HMGB1 detected in HIV-1 exposed pDCs was the consequence of its release by dead cells. We report that inhibition of HMGB1 activity in NK-pDC co-cultures prevented pDCs maturation and IFN-α release, consistent with the observation that HMGB1 translocation from the nucleus to the extracellular environment of TLR9-activated pDCs is required for pDC maturation and IFN-α secretion [38]. Despite HMGB1 release in NK-pDCs co-culture, it did not modulate cell surface expression of maturation markers such as CD83, CD86 and CD40 on pDCs (S5 Fig). This is consistent with findings suggesting that two distinct pathways induce pDC maturation and the release of soluble mediators by pDCs harbouring an immature phenotype [12]. However, high concentration of HIV-1 was sufficient to induce full maturation/activation of pDCs and IFN-α production, suggesting that intracellular TLR signalling by HIV-1 is required for HMGB1-mediated phenotypic maturation and activation of pDCs. The bioactivity of extracellular HMGB1 is regulated by tight interactions with soluble effectors (cytokines and chemokines) [90] or through chemical modification of the three conserved redox-sensitive cysteines (C23, C45, and C106) [34]. Since HMGB1 release in NK-pDC co-culture occurs in the context of the release of many other inflammatory cytokines and chemokines, we hypothesize that HMGB1 activity may be regulated by this microenvironment. The identification of molecules potentially involved in HMGB1 activity in the context of NK-pDC crosstalk is currently under investigation.

Several studies showed that pDCs and mDCs exhibit distinct and complementary functions during immune responses against pathogens. Accordingly, we have previously reported that HIV-1 could not by itself induce the maturation of mDC [43] in the absence of NK cells, whereas in the present study we showed that HIV-1 efficiently induced the maturation of pDCs in a dose-dependent manner. Since the maturation process leads to an increased capacity of cells to communicate with others immune cells by enhancing the expression of co-stimulatory receptors and soluble inflammatory molecules, our finding support the hypothesis that pDCs are the first innate cells sensing the virus and initiating the immune response.

Circulating levels of HMGB1 are elevated during the course of HIV-1 infection [42] and positively associated with high viral load [91]. HMGB1 can be passively released by virus-infected cells including primary CD4 T cells infected with HIV-1, and this was associated with both necrotic and apoptotic cell death [92]. HMGB1 can also be released by non-infected apoptotic CD4 T cells that die through a bystander killing process, which is mainly induced by extracellular HIV-1-encoded proteins and by HIV-1-associated chronic immune activation [93]. Increased circulating HMGB1 levels detected in progressive HIV-1 infection, combined with microbial products (such as LPS) and TLR ligands, may contribute to gut inflammation and subsequent microbial translocation, suggested to have an important role in HIV pathogenesis [94]. In recent studies using and *ex-vivo* model of NK-mDC crosstalk, we suggested the contribution of HMGB1 to HIV persistence in mDCs through the upregulation of two apoptosis inhibitors (cFLIP and c-IAP2) in infected mDC, which rendered them strongly resistant to NK killing [33]. Blocking HMGB1 with specific antibodies restored the susceptibility of infected DCs to NK killing, and similar effect was observed knocking down c-FLIP or c-IAP2 by siRNA [33] [57]. In the present study, we found that HMGB1 had a pivotal role on the expression of mTRAIL on both HIV-1-exposed pDC and NK cells, and it required the production of IFN-α by virus-exposed pDCs. This new function of HMGB1 is compatible with previous observations showing that HMGB1 interaction with its receptor RAGE on the surface of pDCs leads to TLR9-dependent IFN-α release [68], and that IFN-α released by HIV-1 activated pDCs turn them into TRAIL-expressing killer pDCs [95]. Herein, we report that circulating sTRAIL levels were increased in viremic HIV-1^+^ patients, and strongly correlated with circulating HMGB1-specific antibodies and IP-10 levels, arguing for an interdependency between HMGB1, IFN-α and TRAIL that is suggested by our *ex-vivo* model of NK-pDC interaction (Fig 8).

The positive and strong correlation between circulating sTRAIL and HIV-1 proviral DNA is a new finding. It suggests that sTRAIL is a marker of HIV latency in CD4 T cells. Notably, our results are strengthened by studies suggesting that TRAIL contribution to HIV persistence in CD4 T cells from infected patients occurs through the development of CD4 T cell resistance to TRAIL-mediated apoptosis. Indeed, resistance to TRAIL-induced apoptosis may occur through the production of a novel TRAIL splice variant, which can be found in the plasma from infected patients, and which preferentially binds TRAIL-R2, thus preventing proapoptotic TRAIL signaling [96].

In conclusion, our study reports that the concentration of HIV-1 is critical to sustain the functional activation of both pDCs and NK cells. Low levels of HIV-1 were found to mediate the production of inflammatory Th1 cytokines only when pDCs cells were interacting with NK cells. Considering that a very low number of virus particles cross the genital mucosa [97], our observations suggest an important role of NK-pDC crosstalk during the first hours following mucosal infection. At high virus concentration, phenotypic and functional pDC maturation occurred and their cytotoxic function was induced. Regarding NK cells, the induction of their cytotoxic function required their crosstalk with pDCs previously exposed to HIV-1. Several reports showed that the transmitted virus undergoes an amplification step in mucosal tissues before its systemic dissemination. For the design of novel prevention strategies aimed at blocking infection after mucosal HIV-1 exposure, it would be interesting to monitor and to modulate *in vivo* the impact of early activation of NK-pDC cooperation on HIV-1 mucosal transmission and dissemination.

## Materials and Methods

### Characteristics of blood donors

67 HIV-1 infected patients were tested, included in the Nadis cohort, a large prospective French cohort of HIV-infected patients [98]. They were above 18 years of age, with a median CD4 cell count of 499 /μL (IQR 370–764), a median nadir CD4 cell count of 272 /μL (IQR 126–351), and a median CD8 cell count of 832 /μL (IQR 594–1144). 80% of the patients were on combined antiretroviral therapy (c-ART) and the median time on current treatment was 2 years. 57% had undetectable viral load (< 1.6 log_10_), median plasma HIV-1 RNA was 1.6 log_10_ cp/ml (IQR 1.6–2.6) and median HIV-1 proviral DNA was 2.18 log_10_ cp/10^6^ cells (IQR 1.66–2.71). The Ethics Committee in Montpellier (France) approved this study and all subjects gave informed consent prior to screening and enrollment. A control group of healthy adult donors (n = 12) was included. Blood was obtained through the EFS (Etablissement Français du Sang) in the setting of EFS-Institut Pasteur Convention. A written informed consent was obtained for each donor to use the cells for clinical research according to French law. Our study was approved by IRB, external (EFS Board) as required by French law and internal (Biomedical Research Committee Board, Institut Pasteur) as required by Institut Pasteur.

### Isolation and preparation of pDCs and NK cells

Peripheral Blood Mononuclear Cells (PBMCs) were separated from the blood of healthy adult donors on a Ficoll-Hypaque density gradient. pDCs were isolated from fresh PBMCs using the Human Plasmacytoid DC Negative Isolation Kit (StemCell Technologies) according to the manufacturer’s protocol. The enriched cells were assessed for more than 90% purity using the following antibodies: anti-CD123–APC, anti–BDCA-2–PE (Miltenyi Biotec) and anti-CD3-FITC (Becton Dickinson–Pharmingen). pDCs were cultured in RPMI 1640 (Invitrogen, Gaithersburg, MD, USA) containing 10% FCS and 1% penicillin-streptomycin at 37°C in a humidified 5% CO^2^ chamber according to protocol.

CD56^+^ NK cells were isolated by negative selection from fresh PBMCs using the «EasySep NK depletion Kit» (StemCell Technologies). NK cell fraction (CD3^−^CD56^+^) was more than 95% pure, as assessed by flow cytometry (FACScalibur, BD) using FITC-conjugated anti-CD3 and APC-conjugated anti-CD56 antibodies. Contamination with myeloid cells, assessed with FITC-conjugated anti-CD14 antibodies, was consistently less than 1%. Purified NK cells were activated by a combination of PHA (10 μg/ml) (Sigma) and rhuIL-2 (10 μg/ml) (referred as aNK cells), before launching NK-DC coculture experiments.

### Apoptosis measurement

pDC survival was determined with the intracellular Bcl-2 staining, as described previously [99]. Briefly, cultured cells were first stained with CD123 and 20 μg/mL nuclear dye 7-amino-actinomycin D (7-AAD; Sigma-Aldrich) for 30 minutes at 4°C. Cells were then fixed with paraformaldehyde 4% for 20 minutes. To permeabilize cells, perm/wash buffer (BD Biosciences) was used before the intracellular staining with Bcl-2-FITC (clone 124, Dako Inc.). Surviving pDC were identified as CD123+ BCL2^med/high^ 7-AAD^neg^ cells.

### Exposure of pDCs to HIV-1 and cocultures with aNK cells

Virus stock was prepared by amplification of R5-HIV-1_BaL_ on Monocytes-Derived Macrophages (MDM). Viral stock was then clarified by centrifugation prior to determination of HIV-1 p24 concentration. CCR5-tropic Ad5 HIV-1 preparations were treated with DNase I (Takara) in the presence of 10 mM MgCl2 at 37°C for 30 min and then untracentrifuged (17,000 g for 1 hour). Aliquots were stored at -80°C. Freshly purified pDCs were incubated 3 h with various concentrations of HIV-1 (1, 10 or 20 ng p24/ml). pDCs were then plated in 96-well culture plates at 1:5 NK:pDC ratio (2x10^5^ NK:10^6^ pDC) and incubated for 24 hours at 37°C in a 5% CO2 atmosphere. In some experiments, rh-HMGB1 (1 μg/ml) (HMGBiotech srl, Milano, Italy), rabbit anti-HMGB1 Abs (10 μg/ml) (Abcam, Cambridge, UK), Glycyrrhizin (10 μg/ml) or N-ethyl-pyruvate (10 μM) were added at initiation of the coculture. As positive control, pDCs were activated with ODN 2006 (CpG) at 3 μg/ml (InvivoGen, USA). As negative control, pDCs were cultivated in the presence of GpC at 3 μg/ml (InvivoGen, USA), LPS at 10 μg/ml (Sigma-Aldrich) or trimeric CD40L at 500 ng/ml (Sigma-Aldrich).

### Cell surface staining and flow cytometry analyses

The phenotype of pDCs was determined with the following primary mAbs and the appropriate isotype controls (from BD Biosciences, San Jose, CA): CCR5 (clone 2D7), CXCR4 (clone 12G5), CD40-APC (clone 5C3); HLA-DR-APC (clone L243); CCR7-FITC (clone 3D12), CD83-APC (clone HB15e), CD86-APC (clone 2331) and TRAIL-PE (clone RIK-2). CD4 (clone 13B8.2) was purchased from Beckman Coulter. Cells were stained for 30 minutes at 4°C, washed twice in PBS/BSA/NaN3 (0.5% BSA, 0.01% NaN3) and fixed with 1% PFA. For intracellular staining, cells were fixed with 4% PFA, permeabilized using 0.5% BSA, 0.01% NaN3, 0.5% Saponin buffer, stained for 20 minutes at room temperature with FITC-labeled anti-HMGB1 pAbs (ABCAM). At least 5,000 events were acquired using a FACScalibur flow cytometer (BD Biosciences), and stained cells were analysed using FlowJo software (Tree Star, Inc., Ashland, OR). DC survival was assessed using the 7-AAD assay, as described previously [99]. When phenotypic characterization of pDCs was performed in NK-DC cocultures, NK cells were excluded through the gating of CD56^neg^ cells. Surviving pDCs were identified as CD56^neg^ 7-AAD^neg^ cells. When phenotypic characterization of NK cells was performed in NK-DC cocultures, NK cells were gated through their expression of CD56 marker.

### TRAIL and HMGB1 localization by three-dimensional (3D) microscopy

pDCs were cultured overnight in medium (unstimulated), stimulated with CpG (3 μg/ml) or exposed to HIV-1 at 1 ng/ml. pDCs were then plated on poly-l-lysine (Sigma)–coated slides and fixed in 4% paraformaldehyde, quenched with 0.1 M glycine. pDCs were co-stained with mouse anti-TRAIL (clone RIK-2, eBioscience) and Alexa 488–labeled anti-HMGB1 (Abcam) antibodies in permeabilizing buffer containing 1% saponin. TRAIL staining was revealed using a secondary Alexa 547- goat anti-mouse IgG (Jackson ImmunoResearch). Nuclei were stained with DAPI (Molecular Probes). Mounted slides were scanned with a Nikon Eclipse 90i Upright microscope (Nikon Instruments Europe) and were subsequently deconvoluted (Meinel algorithm) and analyzed using Metamorph (MDS Analytical Technologies). Analysis was performed using a 3D viewer from ImageJ. Forty stacks were compiled to make a 3D view of the cells.

### IFN-α detection and inhibitors

pDCs were stimulated for 24 h with ODN 2216 (CpG-A) or ODN 2006 (CpG-B) at 3 μg/ml (InvivoGen, USA). In some experiments, IFN-α release was inhibited with CB (Sigma) at 1 μM. For experiments aiming at neutralizing IFN-α activity, pDCs were incubated for 24 h with antibodies specific for human IFN-α (clone MMHA-1) or human IFN-α/βreceptor (Clone MMHAR-2) (both from PBL Assay Science) at 1 μg/ml. IFN-α was quantified in culture supernatants using the Human IFN-Alpha ELISA Kit (PBL Interferon Source, Piscataway, NJ), according to manufacturer's instructions.

### Assessment of circulating sTRAIL and HMGB1-specific antibody concentrations

Circulating sTRAIL concentration was determined with the human CD253 ELISA kit from Diaclone SAS (Besançon, France). Concentrations of the whole range of circulating anti-HMGB1 IgG antibodies were determined with an in house quantitative ELISA assay that enabled the detection of both residual and complexed HMGB1-specific antibodies. 96-well plates were coated overnight at 4°C with recombinant HMGB1 (HMGBiotech, HM-115) in PBS. Simultaneously, coating of serial dilutions of the calibrator human serum IgG (Sigma) was performed, as previously reported [100]. After washing the plates, unbound sites were blocked with PBS/2% (w/v) BSA. Since anti-HMGB1 antibodies were reported to bind HMGB1 in serum [69], we dissociated these complexes with glycine before titration of the antibodies. Treated samples were then immediately diluted and dispensed in coated plates. Goat anti-human IgG alkaline phosphatase-conjugated antibodies were added, and detection of HMGB1-specific antibodies was performed after incubation with 100μl pNPP substrate. Their concentration was calculated according to the standard curve obtained from standard immunoglobulin solution absorbance by Ascent software, ThermoElectrocorp. The data are expressed in ng/ml of antibodies detected.

### Quantification of cytokines by Luminex

Culture supernatants were analysed using the Human Cytokine Milliplex Kit (Millipore Corporation, Billerica, MA) and the Luminex LX100 (Luminex Corporation, Austin, TX) according to manufacturer’s instructions. The cytokines and chemokines analysed were as follows: IFN-α, TNF-α IL-6, IL-13, IFN-γ, IL-12p40, IL-12p70, IL-10, IL-15, IL-1α, IL-2, IL-3, macrophage-derived chemokine (MDC), monocyte chemoattractant protein 1 (MCP1), MCP3, IFN-γ-inducible protein 10 (IP-10), macrophage inflammatory protein 1α (MIP-1α, MIP-1β, regulated on activation, normal T-cell expressed and secreted (RANTES), growth-regulated oncogene (GRO), granulocyte-macrophage colony stimulating factors (GM-CSF) and Eotaxin. Samples were assayed in duplicate. At least 75 events were acquired for each analyte. Values above or below the standard curves were replaced by the lowest or the highest concentrations measured. For some experiments, flow data were formatted with Pestle v1.6.2 software (Mario Roederer, Vaccine Research Centre, National Institute of Allergy and Infectious Diseases, National Institutes of Health) to facilitate the use of SPICE v5.2.

### Statistical analysis

Statistical analysis was performed using GraphPad Prism version 5.00 (GraphPad Software, San Diego, CA). The data are presented as arithmetic mean ± SD and were compared using Mann-Whitney test and Wilcoxon matched pairs test as appropriate. P-values <0.05 were considered to be significant.

## Supporting Information

S1 Fig Phenotypic characterization of immature and mature pDCs. (A): pDCs were activated for 24h with CpG (3μg/ml), LPS (10 μg/ml), trimeric CD40L (500 ng/ml) or incubated in culture medium (NS). GpC (3μg/ml) was used as a negative control of pDCs maturation. Forward Scatter (FSC) and side-scatter (SSC) parameters were used to discriminate mature pDCs (red) from immature pDCs (blue) under the indicated conditions of stimulation. (B): The expression of the maturation markers CCR7, CD40, CD86, HLA-DR, CD83 was analysed on the surface of FSC^low^ (blue) and FSC^high^ (red) pDCs populations. The percentage of expression of each marker, and mean fluorescence intensity (MFI) for HLA-DR staining, are indicated. Results from one representative experiment out of at least three experiments conducted with different primary cell preparations are shown.
(TIF)

S2 Fig Proinflammatory cytokines and chemokines released by activated NK cells, pDCs and NK-pDC cocultures. Cytokines and chemokines content were quantified by MAP technology in 24 h cell-free culture supernatants of activated NK cells (aNK cells), pDCs cultivated alone or in the presence of aNK cells. The mean ± SD of at least three independent experiments is shown.
(TIF)

S3 Fig Expression of TRAIL at the surface of HIV-1-exposed aNK cells. Activated NK cells (aNK cells) were exposed to CpG (3 μg/ml), increasing concentrations of HIV-1, or incubated in culture medium (NS) for 24h. Membrane TRAIL (mTRAIL) expression on aNK cells was monitored by flow cytometry. Results from one representative experiment out of three experiments conducted with different primary cell preparations are shown.
(TIF)

S4 Fig Impact of HIV-1 on the survival of pDCs. pDCs were exposed to HIV-1 (1 and 20 ng/ml) for 24h or incubated in culture medium (NS). pDCs were intracellularly stained with Bcl-2 antibody. Living pDCs were identified as CD123^Pos^ Bcl2^high^ (red) or CD123^Pos^ Bcl2^med^ cell populations. Histogram (right side) shows the mean ± SD of five independent experiments,
(TIF)

S5 Fig Impact of aNK cells on the phenotypic maturation of pDCs. pDCs were cultured for 24 h in the presence or not of aNK cells. Stimulation with CpG (3 μg/ml) was used as positive control. (A): Forward Scatter (FSC) and side-scatter (SSC) parameters were used to discriminate mature pDCs (red) from immature pDCs (blue) under the indicated conditions of stimulation. (B) Phenotypic characterization of NK-interacting pDCs. The expression of maturation markers CD83, CD80, CD86, HLA-DR and chemokine-receptor CCR7 was analysed by flow cytometry on gated CD123^+^ pDCs. These results are representative of at least three different experiments conducted with primary cells from distinct donors.
(TIF)

S6 Fig HIV-1_Ad5_ triggers the release of proinflammatory cytokines and chemokines by pDCs. Cytokines and chemokines content were quantified by MAP technology in 24 hour cell-free culture supernatants of pDCs incubated in medium (uninf) or exposed to high concentrations of purified CCR5-HIV strain Ad5 (20 ng/ml p24). The mean values ± SD of four independent experiments are shown.
(TIF)
