# Supplementary figures and images for: HMGB1 Is Involved in IFN-α Production and TRAIL Expression by HIV-1-Exposed Plasmacytoid Dendritic Cells: Impact of the Crosstalk with NK Cells
Source: PLoS Pathog. 2016 Feb 12;12(2):e1005407. doi: 10.1371/journal.ppat.1005407 (PMC4752468; doi:10.1371/journal.ppat.1005407)

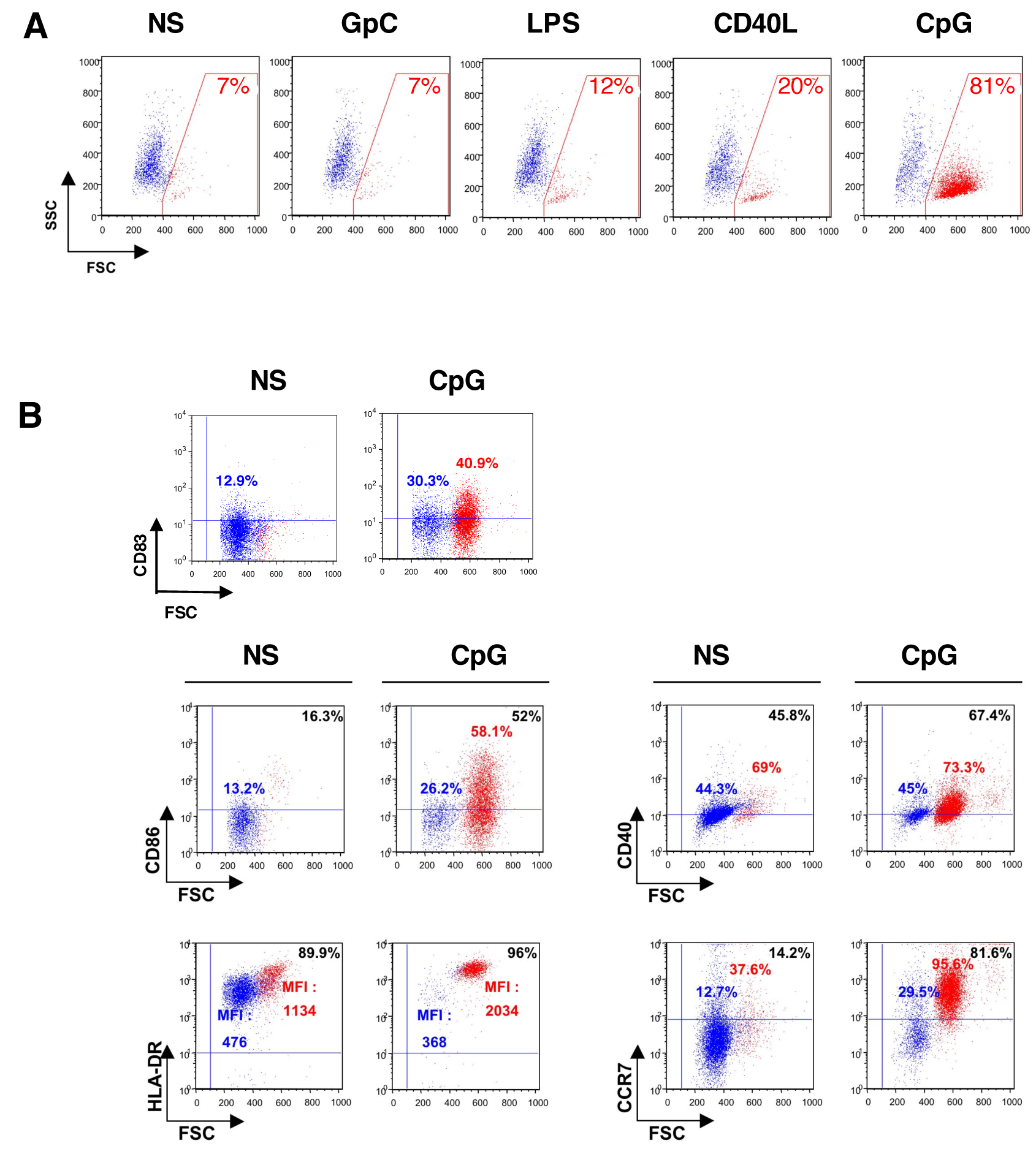

Supplement: S1 Fig — (A): pDCs were activated for 24h with CpG (3μg/ml), LPS (10 μg/ml), trimeric CD40L (500 ng/ml) or incubated in culture medium (NS). GpC (3μg/ml) was used as a negative control of pDCs maturation. Forward Scatter (FSC) and side-scatter (SSC) parameters were used to discriminate mature pDCs (red) from immature pDCs (blue) under the indicated conditions of stimulation. (B): The expression of the maturation markers CCR7, CD40, CD86, HLA-DR, CD83 was analysed on the surface of FSClow (blue) and FSChigh (red) pDCs populations. The percentage of expression of each marker, and mean fluorescence intensity (MFI) for HLA-DR staining, are indicated. Results from one representative experiment out of at least three experiments conducted with different primary cell preparations are shown. (TIF) [file ppat.1005407.s001.tif]

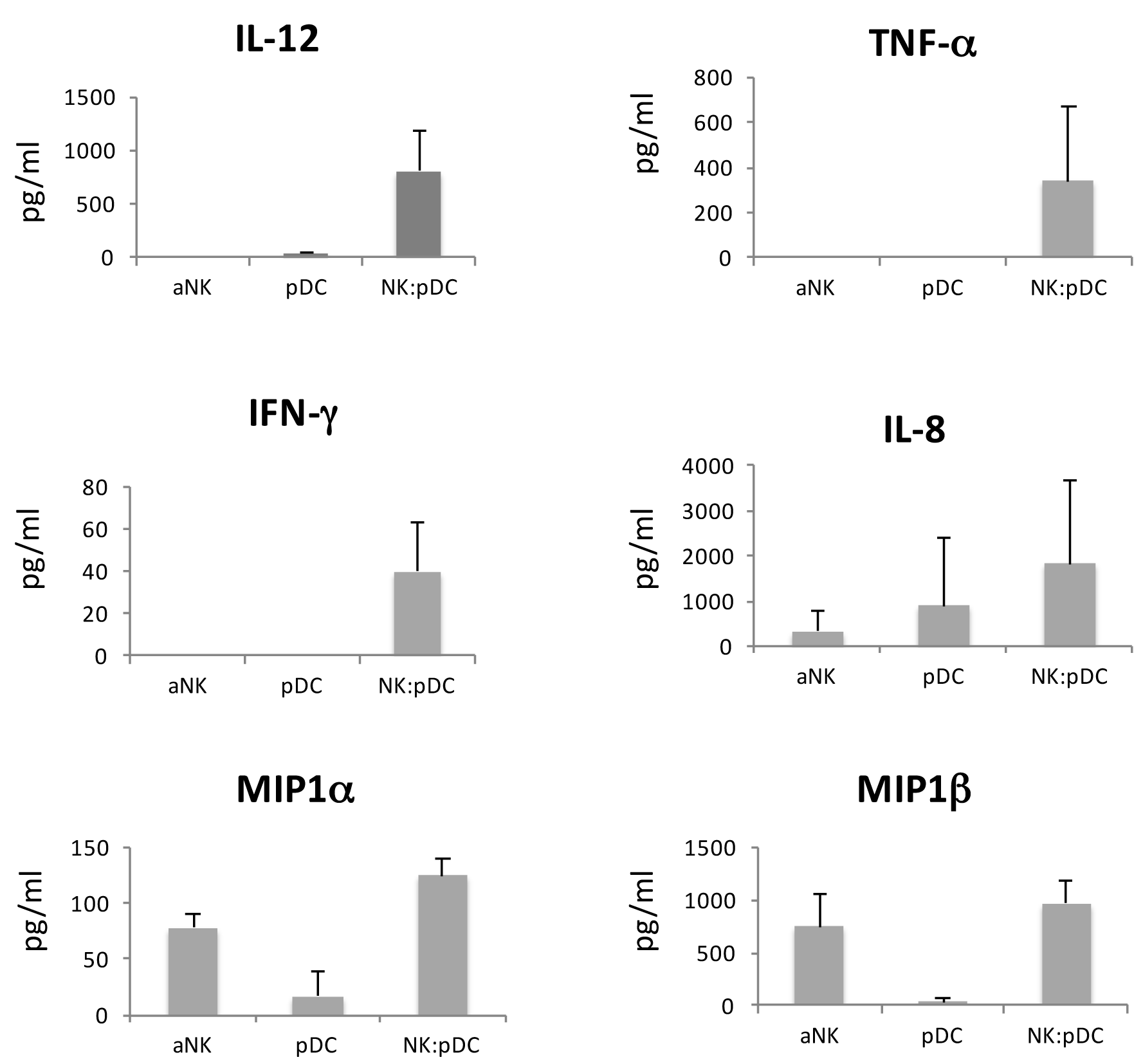

Supplement: S2 Fig — Cytokines and chemokines content were quantified by MAP technology in 24 h cell-free culture supernatants of activated NK cells (aNK cells), pDCs cultivated alone or in the presence of aNK cells. The mean ± SD of at least three independent experiments is shown. (TIF) [file ppat.1005407.s002.tif]

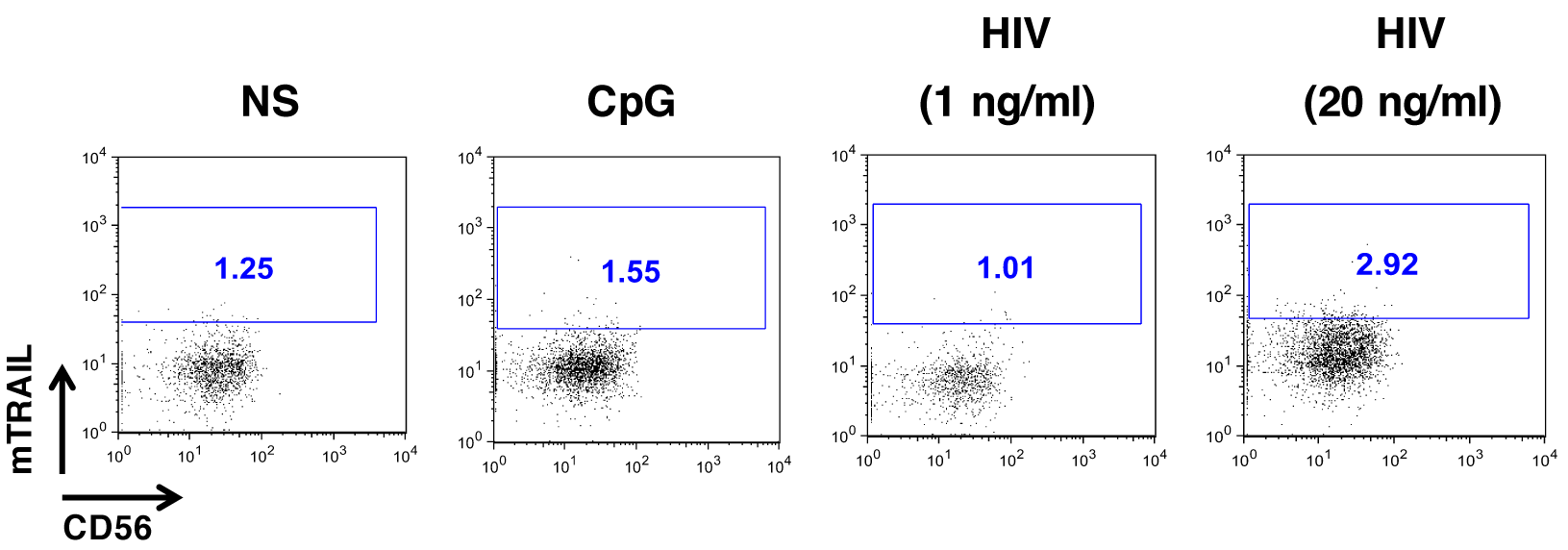

Supplement: S3 Fig — Activated NK cells (aNK cells) were exposed to CpG (3 μg/ml), increasing concentrations of HIV-1, or incubated in culture medium (NS) for 24h. Membrane TRAIL (mTRAIL) expression on aNK cells was monitored by flow cytometry. Results from one representative experiment out of three experiments conducted with different primary cell preparations are shown. (TIF) [file ppat.1005407.s003.tif]

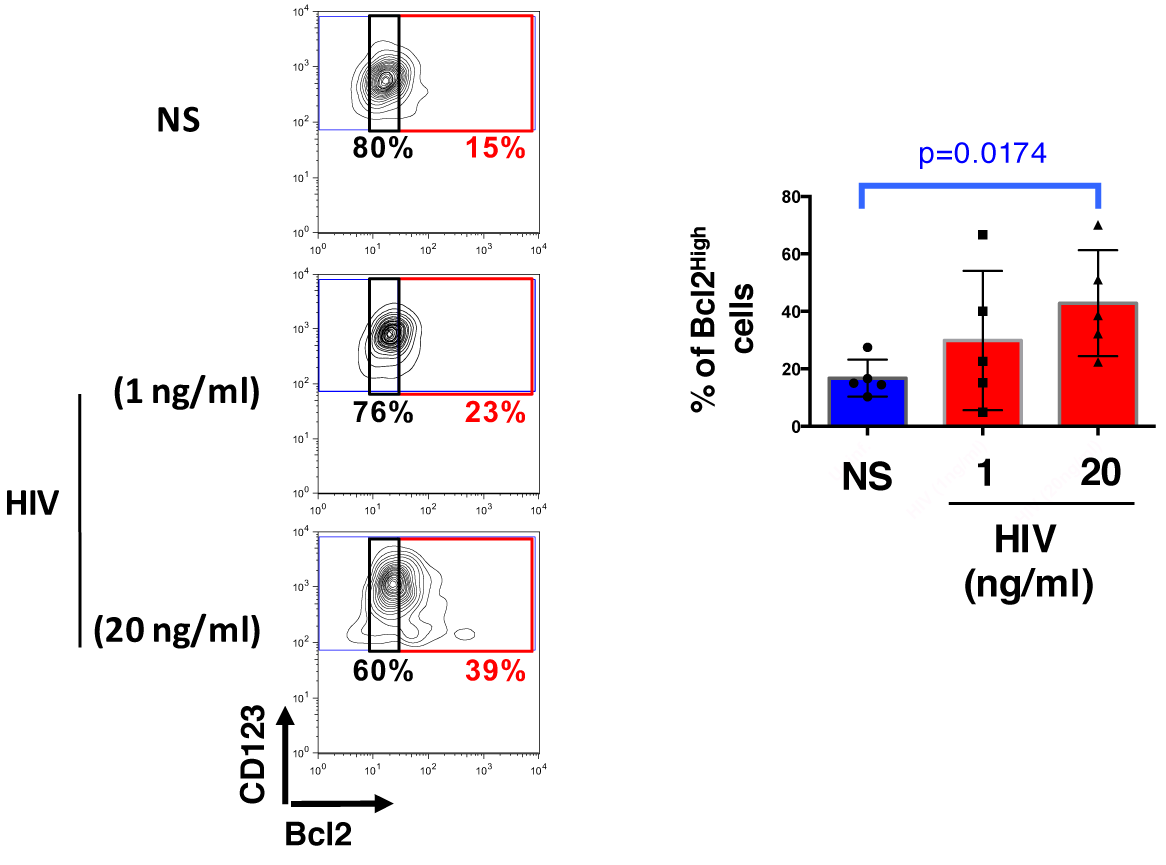

Supplement: S4 Fig — pDCs were exposed to HIV-1 (1 and 20 ng/ml) for 24h or incubated in culture medium (NS). pDCs were intracellularly stained with Bcl-2 antibody. Living pDCs were identified as CD123Pos Bcl2high (red) or CD123Pos Bcl2med cell populations. Histogram (right side) shows the mean ± SD of five independent experiments, (TIF) [file ppat.1005407.s004.tif]

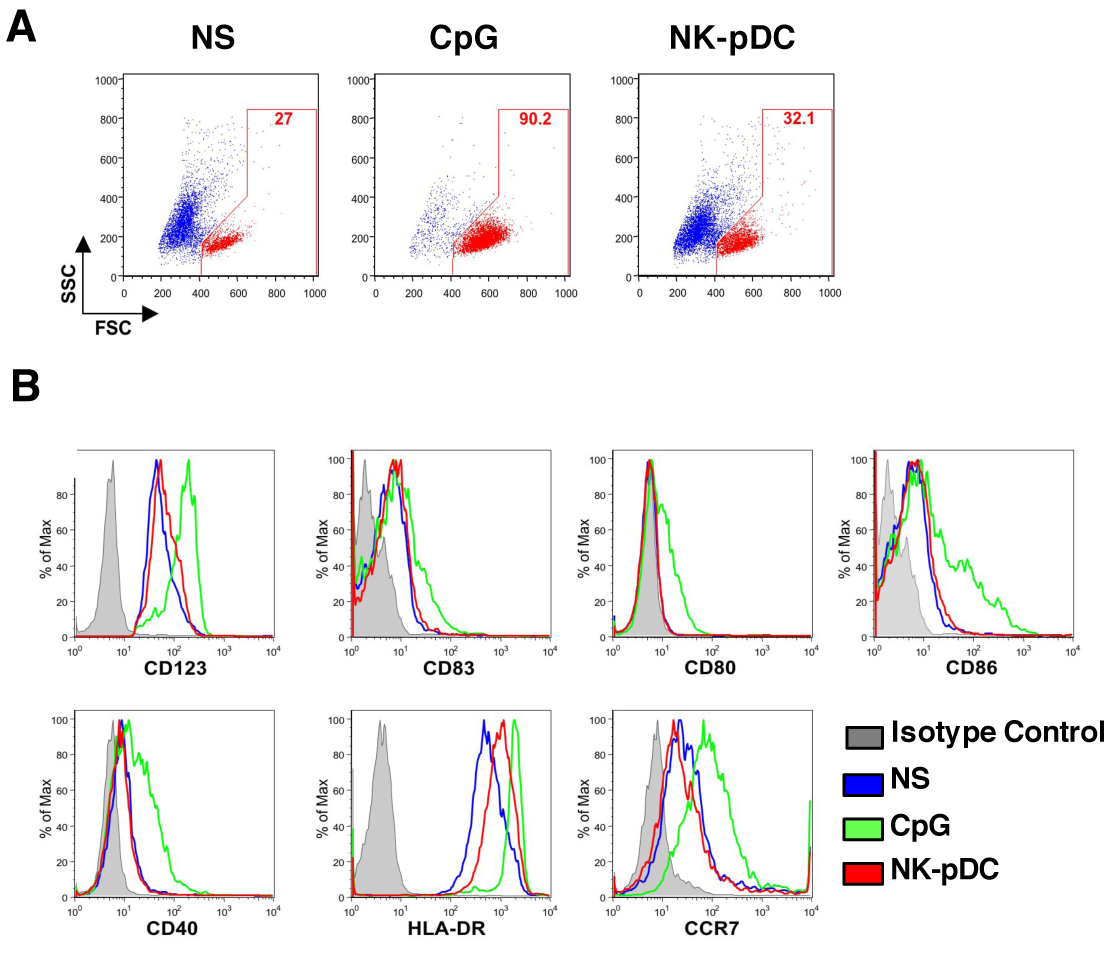

Supplement: S5 Fig — pDCs were cultured for 24 h in the presence or not of aNK cells. Stimulation with CpG (3 μg/ml) was used as positive control. (A): Forward Scatter (FSC) and side-scatter (SSC) parameters were used to discriminate mature pDCs (red) from immature pDCs (blue) under the indicated conditions of stimulation. (B) Phenotypic characterization of NK-interacting pDCs. The expression of maturation markers CD83, CD80, CD86, HLA-DR and chemokine-receptor CCR7 was analysed by flow cytometry on gated CD123+ pDCs. These results are representative of at least three different experiments conducted with primary cells from distinct donors. (TIF) [file ppat.1005407.s005.tif]

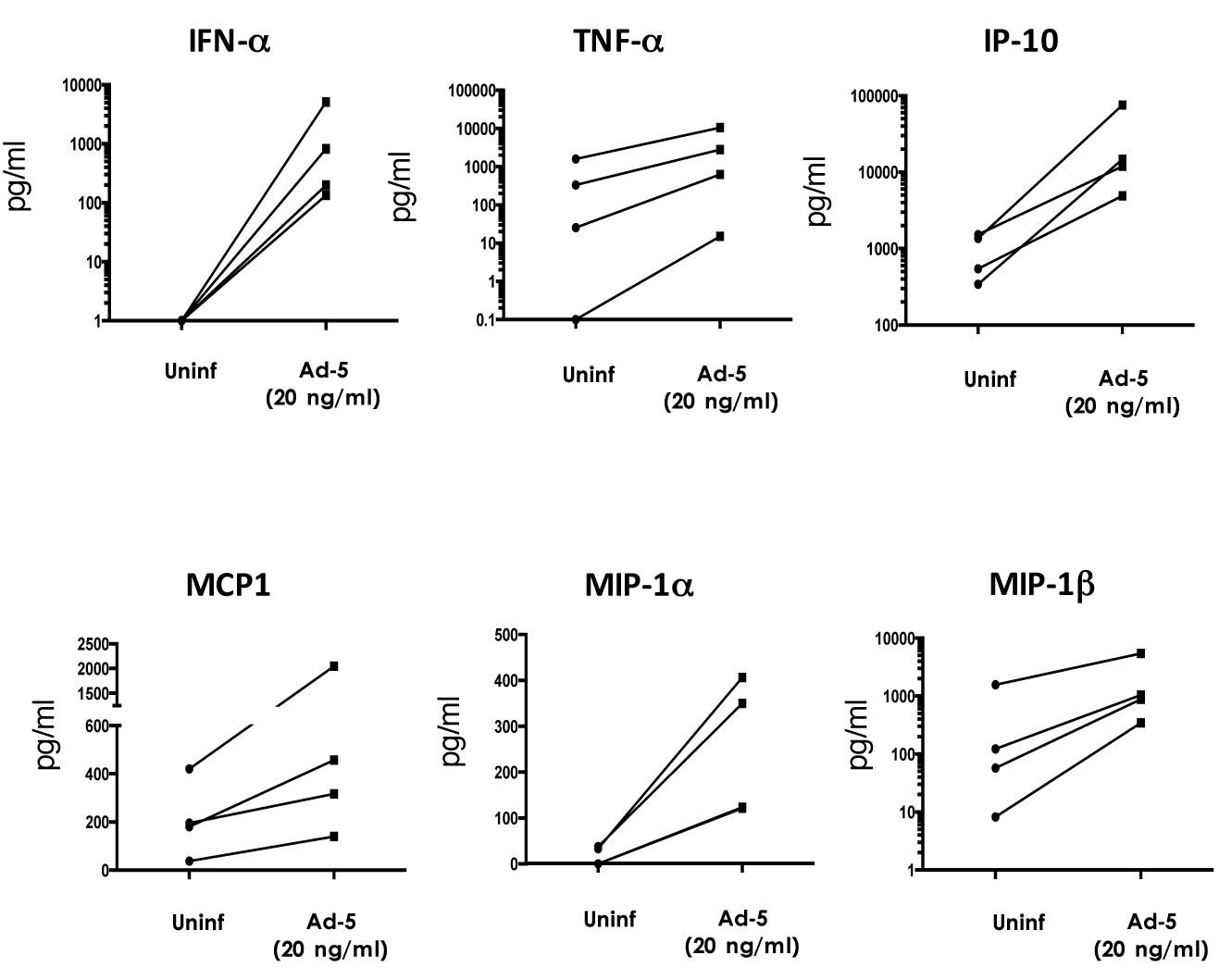

Supplement: S6 Fig — Cytokines and chemokines content were quantified by MAP technology in 24 hour cell-free culture supernatants of pDCs incubated in medium (uninf) or exposed to high concentrations of purified CCR5-HIV strain Ad5 (20 ng/ml p24). The mean values ± SD of four independent experiments are shown. (TIF) [file ppat.1005407.s006.tif]
